# A Zebrafish Model of Neurotoxicity by Binge-Like Methamphetamine Exposure

**DOI:** 10.3389/fphar.2021.770319

**Published:** 2021-11-22

**Authors:** Juliette Bedrossiantz, Marina Bellot, Pol Dominguez-García, Melissa Faria, Eva Prats, Cristian Gómez-Canela, Raul López-Arnau, Elena Escubedo, Demetrio Raldúa

**Affiliations:** ^1^ Institute for Environmental Assessment and Water Research (IDAEA-CSIC), Barcelona, Spain; ^2^ Department of Analytical and Applied Chemistry (Chromatography Section), School of Engineering, Institut Químic de Sarrià-Universitat Ramon Llull, Barcelona, Spain; ^3^ Research and Development Center (CID-CSIC), Barcelona, Spain; ^4^ Department of Pharmacology, Toxicology and Therapeutic Chemistry, Pharmacology Section and Institute of Biomedicine (IBUB), Faculty of Pharmacy, University of Barcelona, Barcelona, Spain

**Keywords:** methamphetamine neurotoxicity, zebrafish model, behavior, neurochemicals, gene expression

## Abstract

Hyperthermia is a common confounding factor for assessing the neurotoxic effects of methamphetamine (METH) in mammalian models. The development of new models of methamphetamine neurotoxicity using vertebrate poikilothermic animals should allow to overcome this problem. The aim of the present study was to develop a zebrafish model of neurotoxicity by binge-like methamphetamine exposure. After an initial testing at 20 and 40 mg/L for 48 h, the later METH concentration was selected for developing the model and the effects on the brain monoaminergic profile, locomotor, anxiety-like and social behaviors as well as on the expression of key genes of the catecholaminergic system were determined. A concentration- and time-dependent decrease in the brain levels of dopamine (DA), norepinephrine (NE) and serotonin (5-HT) was found in METH-exposed fish. A significant hyperactivity was found during the first hour of exposure, followed 3 h after by a positive geotaxis and negative scototaxis in the novel tank and in the light/dark paradigm, respectively. Moreover, the behavioral phenotype in the treated fish was consistent with social isolation. At transcriptional level, *th1* and *slc18a2* (*vmat2*) exhibited a significant increase after 3 h of exposure, whereas the expression of *gfap*, a marker of astroglial response to neuronal injury, was strongly increased after 48 h exposure. However, no evidences of oxidative stress were found in the brain of the treated fish. Altogether, this study demonstrates the suitability of the adult zebrafish as a model of METH-induced neurotoxicity and provides more information about the biochemical and behavioral consequences of METH abuse.

## Introduction

Methamphetamine (METH) is a highly addictive psychostimulant drug affecting both the dopaminergic and serotonergic systems in the central nervous system (CNS) (Kobeissy et al., 2012). Some of the effects of METH related with CNS-stimulation include euphoria, increased alertness, aggressiveness, intensified emotions and altered self-esteem (Davidson et al., 2001). Some METH abusers binge on the drug for days in order to prolong euphoria (Shabani et al., 2019). METH binge is characterized by the administration of large doses of this drug, at short inter-dose durations (every 2 h), over several days (typically 1–3 days). This abuse pattern results in nearly steady-state plasma levels of METH during most of the binge period (Davidson et al., 2001; Shabani et al., 2019). Acute and chronic exposure to toxic doses of METH induces unpleasant CNS symptoms such as anxiety, depression, hallucinations, psychosis, and seizures, as well as uncontrollable hyperthermia (Davidson et al., 2001; Darke et al., 2008). Whereas central dopaminergic system seems to mediate the changes in mood, excitation level and motor movement, serotonergic system may also contribute to the METH-related mood changes, psychosis, and aggressiveness (Albertson et al., 1999).

The neurotoxic effects of the METH binge administration regime, with constant high levels of METH in the CNS, have been extensively investigated (Shin et al., 2021). In fact, it is widely known that METH is able to induce the release of monoamine neurotransmitters [dopamine (DA), serotonin (5-HT), and norepinephrine (NE)] from nerve endings (Sulzer et al., 1995; Jones et al., 1998; Sitte et al., 1998; Fleckenstein et al., 2009). The mechanism apparently involves the initial redistribution of these neurotransmitters to the cytoplasm from the synaptic vesicles via the vesicular monoamine transporter 2 (VMAT2) and then the reverse transport of neurotransmitters into the synapses via specific plasma membrane transporters (DAT, NET, SERT) (Kish, 2008). The released DA will react with molecular oxygen to form quinones and semiquinones, as well as reactive oxygen species (ROS), finally resulting in oxidative stress generation and injury in dopaminergic terminals (Kita et al., 2009). Neurotoxic effects after high doses of METH in rodents include, therefore, long-lasting depletion in the striatal content of DA and its metabolites (Ricaurte et al., 1982), decrease in tyrosine hydroxylase (TH) activity (Ellison et al., 1978), and loss of DATs (Escubedo et al., 1998), a marker of terminal integrity. The persistent loss of DA axons in mice striatum induced by METH has been correlated with DA cell body loss in the substantia nigra pars compacta (SNpc) (Granado et al., 2011; Ares-Santos et al., 2012). However, the mechanisms underlying METH-induced striatal neurotoxicity are complex and still being investigated (Pubill et al., 2003, 2005). Moreover, the hyperthermia associated to acute toxic dosing of METH has been related with changes in the neurochemical profile, oxidative stress and neurodegeneration (reviewed at Davidson et al., 2001). Therefore, hyperthermia is an important confounding factor for understanding of the specific neurotoxic effects induced by METH. Consequently, this leads us to believe that new animal models of METH neurotoxicity could provide interesting new insights.

Zebrafish is an emerging model for studying complex brain disorders, covering the major human neuropsychiatric and neurotoxic syndromes (Kalueff et al., 2014; Stewart et al., 2014). This poikilotherm organism exhibits an overall nervous system organization and neurotransmitter systems similar to humans, responding also in a similar fashion to most of the neurotropic drugs used in human pharmacology. As a result, zebrafish has emerged as a new and powerful model species in translational neuroscience, including in the study of drug abuse-related phenotypes (Stewart et al., 2011; Neelkantan et al., 2013; Kolesnikova et al., 2019, 2021). Although a few studies have analyzed some effects of METH in adult zebrafish (Jiang et al., 2016; Mi et al., 2016; Zhu et al., 2017), most of them were focused on METH addiction and not in assessing neurotoxicity. The development of a model of METH neurotoxicity in this poikilotherm organism should be extremely useful for deciphering the direct effects of METH without the confounding factor of hyperthermia.

In this study adult zebrafish were continuously exposed to 20 or 40 mg/L METH for 48 h in order to achieve high and constant METH levels in CNS, as it is attained in the binge rodent models. Effects on motor activity, anxiety-like behavior, and social behavior have been thoroughly analyzed, as well as changes in the monoaminergic profile and gene expression in the brain of the fish at 3 and 48 h after exposure. Finally, the presence of oxidative stress in the whole brain of the fish was also analyzed after 48 h exposure.

## Materials and Methods

### Animals and Housing

Adult wild-type zebrafish (standard length: 3.5–4.0 cm; weight: 0.62–0.67 g) were obtained from Exopet (Madrid, Spain) and maintained into a recirculating zebrafish system (Aquaneering Inc., San Diego, United States) at the Research and Development Center zebrafish facilities (CID-CSIC) for 2 months before starting the exposures. Fish were housed in 2.8 L tanks (density: 20 fish/tank) with fish water [reverse-osmosis purified water containing 90 mg/L Instant Ocean^®^ (Aquarium Systems, Sarrebourg, France), 0.58 mM CaSO_4_ · 2H_2_O, and 0.59 mM NaHCO_3_] under a 12L:12D photoperiod. The main parameters of the fish water in the housing facilities were: temperature: 28 ± 1°C; pH: 7.6–8.0; conductivity: 700–800 μS/cm; hardness: 120–130 mg/L. Fish were fed twice a day with flake food (TetraMin, Tetra, Germany). All procedures were approved by the Institutional Animal Care and Use Committees at the CID-CSIC (OH 1032/2020) and conducted in accordance with the institutional guidelines under a license from the local government (agreement number 9027).

### Chemicals and Material

Crystalline solid standards of l-tyrosine (Tyr), dopamine hydrochloride (DA), 3,4-dihydroxyphenylacetic acid (DOPAC), homovanillic acid (HVA), l-tryptophan (Trp), 5-hydroxy-l-tryptophan (5-HTP) and serotonin hydrochloride (5-HT) were purchased from Sigma-Aldrich (St. Louis, MO, United States). 3-methoxytyramine hydrochloride (3-MT) was obtained from Merck (Darmstadt, Germany), norepinephrine (NE) was supplied by Tocris Bioscience (Ellisville, United States) and 5-hydroxyindoleacetic acid (5-HIAA) was provided by Toronto Research Chemicals (TRC, Toronto, Canada). Labelled neurotransmitters standards such as 5-HT-d4 HCl, 5-HTP-d4, DOPAC-d5, Trp-1–13C, l-dopa-d3, 5-HIAA-d5 and dl-NE-d6 HCl were obtained from Toronto Research Chemicals (TRC, Toronto, Canada), whereas DA-1,1,2,2-d4 HCl was provided from Merck (Darmstadt, Germany) and 3-MT-d4 HCl from Sigma-Aldrich (St. Louis, MO, United States). METH hydrochloride was generously provided by Prof. J. Camarasa, Faculty of Pharmacy at University of Barcelona (Spain).

Acetonitrile (ACN) and methanol (MeOH) LC-MS grade were purchased from VWR Chemicals Prolabo (Leuven, Belgium). Formic acid (FA) was provided by Fischer Scientific (Loughborough, United Kingdom), while dimethyl sulfoxide (DMSO), ammonium formate and sodium bicarbonate (NaHCO_3_) were supplied by Sigma-Aldrich (St. Louis, MO, United States). Ultra-pure water was daily obtained through Millipore Milli-Q purification system (Millipore, Bedford, MA, United States).

Stock solutions of all labeled and unlabeled compounds were prepared at 1 mg/ml in MeOH, DMSO or ultra-pure water depending on their solubility. Standard solutions of unlabeled neurotransmitters as well as the mixture of labeled standards (ISM) used as internal standard were prepared at the desired concentration in starting mobile phase solvent.

Working solutions of METH were prepared at 20 and 40 mg/L in fish water (pH 8).

### Stability of Methamphetamine in Water

Working solutions of 20 mg/L (C_1_) and 40 mg/L (C_2_) METH were freshly prepared from solid METH by dissolving it in fish water (pH 8). Fish water was used as control solution (C_0_). Solutions (500 ml) were introduced in beakers and kept in an incubation chamber (POL-EKO APARATURA Climatic chamber KK350, Poland) set to 28.5°C and 12 h light: 12 h dark photoperiod. Sample aliquots (C_0_, C_1_ and C_2_) were picked at 0, 24 and 48 h. Standards of 5, 10, 15, 30, 50 mg/L of METH were used to obtain a calibration line.

Water samples were analyzed using an Acquity UPLC^®^ H-class system coupled to fluorescence detector (Waters, MA). METH elution was achieved in a gradient mode with a BEH C18 column (100 mm × 2.1 mm, 1.7 μm) maintained at 35°C. The mobile phase consisted of H_2_O with 0.1% of FA (solvent A), and acetonitrile (solvent B). The gradient started at 5% of B and increased to 15% B in 1.5 min. Then, solvent B increased to 20% in 4 min, and held for 1 min. Finally, initial conditions were restored in 0.5 min, and held for 3 min resulting in a total run time of 10 min.

Aliquots of 10 µl were injected in both standards and samples, using a flow rate of 200 μl min^−1^. The fluorescence detector excitation/emission wavelengths were 256 and 288 nm. Chromatographic data acquisition and processing were performed using Empower 2 software (Waters, Milford, MA, United States).

### Experimental Procedure

In order to build a METH acute neurotoxicity model in adult zebrafish, the first step was to determine the METH concentration with strongest effects on neurobehavior and neurochemicals profile, but without evidences of systemic toxicity (Experiment 1). Three METH concentrations, 20, 40 and 80 mg/L, were selected for an initial range-finding test, as this range of METH concentrations has been already used in drinking water in some rodent models (Shabani et al., 2016). The highest concentration, 80 mg/L METH, was discarded, as this concentration exhibited a 100% of lethality in only 24 h in the range-finding test. No lethality, however, was found with 20 and 40 mg/L after 48 h exposure. Supplementary Figure S1 shows the experimental design of the two types of experiments performed in this study. Working solutions of 20 and 40 mg/L METH were directly prepared in fish water (pH 8) the day of the experiment. Adult zebrafish (≈50:50 male:female ratio) were randomly selected from the CID-CSIC facilities and exposed for 48 h to 20 or 40 mg/L METH, at 28.5°C and 12L:12D photoperiod. Control fish were maintained in fish water under identical conditions. Experiments were conducted in duplicate or triplicate, in glass tanks containing 500 ml of water with three fish in each. Experimental solutions were renewed at 24 h, 30 min after the first feeding of the day. Tanks were kept in an incubation chamber (POL-EKO APARATURA Climatic chamber KK350, Poland) set to 28.5°C and 12L:12D photoperiod. Fish were euthanized by inducing hypothermic shock in ice-chilled water (2–4°C) and brains for neurotransmitters, gene expression and oxidative stress were immediately dissected and individually stored at −80°C for further analysis.

### Behavioral Testing

Behavioral testing was performed in an isolated behavior room (27–28°C) between 10:00 and 17:00 h. Animals (≈50:50 male:female ratio) were transferred to the behavior room 1 hour before testing began, for acclimation. All fish used in this study were experimentally naïve and the testing was performed in a blind manner. In order to avoid the potential interference of any conspecific alarm cues, water of the experimental tank was changed after each trial.

Locomotor activity was determined after placing shoals of six fish into an experimental tank containing fish water (control group) or 40 mg/L METH (treated group) and video-recorded for 180 min. Two independent trials were conducted (*n* = 12). The distance moved and the percentage of time spent in hypermobility state [when the software detects changes in more than 70% of the pixels identified as the during at least 150 consecutive frames (≥5 s)] by each fish of the shoal was analyzed using Ethovision XT 13.0 social interaction module.

The Novel Tank Test (NTT), used to assess geotaxis (preference for the bottom of the tank), freezing and erratic movements of fish, was performed using an experimental setup previously described (Faria et al., 2018). Videos from each trial were analyzed by Ethovision XT 13.0 (Noldus, Wageningen, Netherlands). First, the front of the tank was divided into two equal virtual zones, top and bottom, and the distance travelled at the top and at the bottom (cm), the time spent in the top (s), latency to top (s), and entrances to the top were determined. Freezing behavior was defined as immobility (identified by the software, when less than 3% mobility) combined with increased opercular movements (visually identified by the observer), for at least 5 s. Additional details are provided in Supplementary Methods.

The Dark-Light Test (DLT), used to determine scototaxis (preference for the dark background) of the fish, was performed using an experimental setup previously described (Faria et al., 2019). Each trial was video-recorded with a GigE camera mounted on the top of the experimental tank. The recorded videos were analyzed by Ethovision XT 13.0, and the time spent in the white zone (s), number of transitions to the white zone and the latency to enter to the white zone (s) were determined. Additional details are provided in Supplementary Methods.

The shoaling test, used to assess social and anxiety-like behaviors, was performed using an experimental setup previously described (Green et al., 2012) Two independent trials were performed, with a shoal size of nine fish per experimental group in each (*n* = 18). Groups of nine zebrafish from the control and METH groups were video-recorded for 6 min in our novel tank (Supplementary Methods), and analyzed using Ethovision XT 13.0 social interaction module. This automated method permits us to detect and track every fish in the group and measure the distance moved and inter-fish distances continuously in order to assess shoal density or the shoaling tendency of each individual. The total distance (cm) as well as the average interfish distance (cm), farthest neighbor distance (cm) and nearest neighbor distance (cm) were calculated.

Finally, the social preference test was adapted from the protocol described by (Carreño Gutiérrez et al., 2019). A single fish was transferred to an experimental tank (20 cm length, 20 cm width, 25 cm height) containing 5 L. The single focal fish was allowed to interact with a stimulus compartment–a fish’s shoal placed into the one-side extern housing tank, or with the non-stimulus compartment–a one-side extern empty housing tank. The focal fish was recorded 6 min (AVI video format, 30 fps) with the uEye Cockpit software (version 4.90; Imaging Development Systems, Germany) controlling the GigE cameras (UI-5240CP-NIR-GL, Imaging Development Systems, Germany) placed in front of the testing tanks. The recorded videos were analyzed with Ethovision XT 13.0 (Noldus, Wageningen, the Netherlands). First, the central experimental arena was divided into three equal size virtual zones: empty, center and conspecific (Supplementary Figure S2). The time spent (s) and the distance moved (cm) by the focal fish, was recorded in each arena.

### Analysis of Neurochemicals by LC-MS/MS

The profile of monoaminergic neurochemicals was determined in the whole brain of controls and fish exposed for 3 and 48 h to 20 and 40 mg/L METH. Extraction of monoamine neurochemicals from zebrafish brain was mainly based in the methodology described by Mayol-Cabré et al. (2020). This extraction procedure required working at 4°C during all the process due to the high degradation of target NTs compounds. Briefly, each zebrafish whole brain was introduced in an Eppendorf tube with 300 µl of cold extractant solvent (90:10 ACN/H_2_O + 1% FA), 50 µl of the ISM solution and three stainless steel beads (3 mm diameter), and were homogenized and grounded using a bead mill homogenizer (TissueLyser LT, Quiagen, Hilden, Germany) programmed at 50 osc/min for 90 s. The supernatant was transferred to a new Eppendorf tube, centrifuged for 20 min at 13,500 rpm in a cold room (4°C), filtered through 0.22 µm nylon filters (Scharlab, Barcelona, Spain) and kept in chromatographic vials at −80°C until the analysis. Supplementary Figure S3 summarizes the zebrafish brain extraction procedure with five different stages and subsequent LC-MS/MS analysis.

LC-MS/MS analysis was performed using an Acquity UPLC^®^ H-Class liquid chromatograph (Waters, Milford, MA, United States) coupled to a triple quadrupole mass spectrometer (Xevo, TQ-S micro, Waters, United States) and equipped with an electrospray ionization source (ESI) able to measure the neurotransmitters under positive electrospray ionization. An Acquity UPLC BEH Amide column (150 mm × 2.1 mm ID, particle size 1.7 μm) provided with an Acquity UPLC BEH Amide pre-column (5 mm × 2.1 mm ID, particle size 1.7 μm) (Waters, Milford, MA, United States) was employed. Column operated at a flow rate of 0.25 ml min^−1^ at 30°C. Aliquots of 10 μl of standard and/or samples were injected at 10 ± 5°C. Mobile phase consisted in Milli-Q water and acetonitrile (H_2_O:ACN) (95:5) containing 100 mM ammonium formate at pH 3.0 (solvent A) and H_2_O:ACN (15:85) containing 30 mM ammonium formate at pH 3.0 (solvent B). pH was adjusted to 3 with FA 80%. The LC gradient used for the chromatographic separation started at 100% B, decreased at 80% B in 4 min, and held for 1 min. From 5 to 7 min, B was increased to 100%. Finally, initial conditions were accomplished in 3 min, resulting in a 10 min total run time. Desolvation gas (N_2_) flow was set to 900 L h^−1^, whereas cone gas (N_2_) flow was performed at 150 L h^−1^. Source temperature was set at 100°C and a capillary voltage of 2.0 kV was applied. Desolvation temperature was 350°C. Acquisition was performed in SRM mode, using the most intense fragment as the quantifier ion, and the second most intense as the qualifier ion. Data were acquired and processed using MassLynx® Software v 4.1 (Waters, Manchester, United Kingdom).

### Analysis of Methamphetamine Levels in Brain

Brains of fish exposed to 40 mg/L METH for 3 or 48 h, were extracted by using the same protocol already described for neurochemicals. Extracts were evaporated to dryness under a gentle stream of nitrogen (N_2_) in a sample concentrator (Techne®, Staffordshire, United Kingdom) and reconstituted with 300 µl of ultra-pure water.

For the analysis of METH in the reconstituted extracts, an Acquity UPLC BEH C18 column (100 mm × 2.1 mm ID, particle size 1.7 μm) provided with an Acquity UPLC BEH C18 pre-column (5 mm × 2.1 mm ID, particle size 1.7 μm) (Waters, Milford, MA, United States) were employed. Elution was performed using a binary mobile phase of MilliQ water with 0.1% FA (A) and acetonitrile (B) in a gradient mode at 0.2 ml/min. The gradient started at 5% B, and held for 0.5 min. Then, it was increased to 30% B in 5.5 min, and held for 1 min. Finally, initial conditions were reached in 1.5 min, and held for 2 min, resulting in a total run time of 10 min. Autosampler injection volume was set at 10 μl, and the samples were maintained at 4°C during the injection sequence. MS conditions were similar to the described for neurochemicals.

### Quality Assurance of Neurotransmitters, Methamphetamine in Brain and Methamphetamine in Water Studies

Extractant solvent (ACN:H_2_O 90:10 + 1% FA) was used to prepare the calibration standards. Linearity was performed over a concentration range from 0.005 to 1 mg/L, using six calibration points. ISM was used as internal labeled standards for extraction and analytical quality control. Target compounds were quantified by internal calibration, except for HVA that was determined using external calibration. The validation of the method was checked with the recovery studies selecting seven replicates, using zebrafish brain samples spiked at 250 ng (QCs) with the neurochemical standard mixture and the ISM. In addition, the matrix effect (ME) was calculated comparing slopes between standard calibration line and matrix calibration line. Instrumental detection limits (IDLs) were determined using the lowest concentration standard solution at 0.005 mg/L that yielded a S/N ratio equal to 3. Method detection limits (MDLs) were calculated using QCs that also produced a S/N ratio equal to 3. Moreover, intra-day precision was assessed by three consecutive injections of 1 mg/L standard solution, while inter-day precision was determined by measuring the same standard solution on four different days. The analysis of solvent blanks showed no carryover effect during the LC-MS/MS analysis in all neurochemicals.

In the study of the levels of METH in zebrafish brain, standards of 0.5 to 1,000 μg/L were prepared in water and used to check linearity. Instrumental detection limits (IDLs) were determined using the 0.5 μg/L standard. Inter-day and intra-day precision was determined by measuring the standard solution of 50 μg/L.

For the study of METH stability in water, standards of 5, 10, 15, 30, and 50 mg/L of METH were used to check the calibration range. External calibration was used for METH determination in the stability study. Instrumental detection limits (IDLs) were determined using the lowest concentration standard solution at 5 mg/L. Intra-day precision was determined by measuring a standard of 5 mg/L three times the same day, and inter-day precision was determined by measuring standard solution of 10 mg/L on three different continuous days.

### Real-Time Polymerase Chain Reaction

Total RNA was extracted from the brain of zebrafish control and exposed to 40 mg/L METH for 3, 24, and 48 h, using Trizol Reagent (Invitrogen Life Technologies, Carlsbad, CA). RNA quality and concentration were determined on a NanoDrop ND-8000 spectrophotometer (NanoDrop Technologies). First strand cDNA was synthesized from 1 μg of total RNA previously treated with DNaseI (Ambion, Austin, TX), using First Strand cDNA synthesis Kit (Roche Diagnostics, Mannheim, Germany) and oligo(dT) according to manufacturer’s instructions.

Real Time qRT-PCR was performed in a LightCycler^®^ 480 Real-Time PCR System with SYBR Green PCR Master Mix (Roche Diagnostics, Mannheim, Germany). Cycling parameters: 95°C for 15 min and 45 cycles of 95°C, 10 s and 60°C, 30 s. A dissociation curve analysis was added to ensure the specificity of the reaction. Eight biological replicates were assayed for each treatment, and three technical replicates were run for each sample.

Primer sequences for the selected genes [tyrosine hydroxylases 1 and 2 (*th1, th2*), dopamine transporter *(dat* or *slc6a3*), vesicular monoamine transporter (*vmat2* or *slc18a2*), dopamine-β-hydroxylase (*dbh*), and glial fibrillary acidic protein (*gfap*)] are listed in Supplementary Table S1. Specificity of primers was previously confirmed by the presence of a single peak in the melting curves of PCR reactions.

The mRNA expression of each target gene was normalized to the housekeeping *ppia2* as reference gene. Relative quantification of mRNA abundance for the selected genes among treatments with respect to the control group were calculated following the ΔΔCt method (Livak and Schmittgen, 2001) deriving fold-change ratios from these values.

### Lipid Peroxidation Determination

Lipid peroxidation was determined in the brain of zebrafish exposed 48 h to METH, by quantifying the levels of malondialdehyde (MDA) according to Faria et al. (2019). Pools of three brains were homogenized at a proportion of 50 mg/ml (tissue weight/buffer volume), in ice cold 0.1 M phosphate buffer with 150 mM KCl and 0.1 mM ethylenediamine-tetraacetic acid, disodium, salt, dihydrate (EDTA) and 0.01% BHT. The brain homogenates were then incubated with 5 mM 1-methyl-2-phenylindole prepared in acetonitrile:methanol (3:1 v/v), 5.55% of HCl at 45°C, for 40 min. Following incubation, absorbance was read at 586 nm and MDA content in each sample was extrapolated from a standard curve of 1,1,3,3-tetramethoxypropane (TMP) treated under similar conditions as samples. The final results were normalized by mg of tissue ww (wet weight) corresponding to each sample and expressed as pmol/mg.

### Data Analysis

Data were analyzed with IBM SPSS v26, and plotted with GraphPad Prism 9.02 for Windows (GraphPad software Inc., La Jolla, CA). Normality was assessed using Shapiro-Wilk test. One-way ANOVA followed by Dunnett’s or Tukey’s multiple comparison test was used to test differences between normally distributed groups, whereas the Kruskal-Wallis test followed by a pairwise comparison using the Bonferroni correction was used to test differences between groups that did not meet parametric assumptions.

## Results

### Stability of Methamphetamine in Fish Water

Supplementary Table S2 shows the measured concentrations and the stability of METH in fish water in our experimental conditions. The measured concentrations, 20.66 ± 0.02 and 40.05 ± 0.11 mg/L, were very close to the nominal values tested in this study, 20 and 40 mg/L. Moreover, no significant degradation of METH with time was found for both 20 mg/L (*H*_(2)_ = 0.605, *p* = 0.739) and 40 mg/L (*H*_(2)_ = 4.356, *p* = 0.113) concentrations. Finally, no signal was observed in fish water control samples, proving that there was no cross-contamination.

### Determining the Methamphetamine Concentration to Build the Acute Neurotoxicity Model

The NTT paradigm was used to determine the effects on anxiety-like behavior (Supplementary Figure S4) in animals treated with the 20 and 40 mg/L METH 48 h after exposure. Both concentrations of METH induced an anxiety-like state characterized by positive geotaxis, with a significant decrease in the time spent in the top of the tank (*H*_(2)_ = 19.030, *p* = 0.00007), the distance moved in this zone (*H*_(2)_ = 23.027, *p* = 9.99 × 10^−6^) and the number of entrances to the top (*H*_(2)_ = 25.295, *p* = 3.21 × 10^−6^). Moreover, the latency to enter to the top increased in the treated animals (*H*_(2)_ = 28.631, *p* = 6.07 × 10^−7^). No differences between the fish exposed to 20 and 40 mg/L METH were found in any of the parameters measured in the NTT (*p* > 0.05 when results were adjusted by the Bonferroni correction).

When the neurochemical profile of the control and treated fish was determined (Supplementary Figure S5), the main effect of METH was a significant decrease in the levels of the monoaminergic neurotransmitters DA (*F*_(2,28_) = 57.523, *p* = 1.21 × 10^−10^), NE (*F*_(2,28)_ = 115.191, *p* = 3.08 × 10^−14^) and 5-HT (*F*_(2,28)_ = 50.063, *p* = 5.67 × 10^−10^). However, no differences between the fish exposed to 20 and 40 mg/L METH were found in the levels of these three neurotransmitters (*p* > 0.05; one-way ANOVA followed by Tukey’s multiple comparison test). Whereas the levels of the DA metabolite, DOPAC, were also significantly decreased by both METH concentrations ((*H*_(2)_ = 15.164, *p* = 0.00051), only 40 mg/L METH was able to decrease the levels of 3-MT (*H*_(2)_ = 10.827, *p* = 0.0044), HVA (*H*_(2)_ = 11.069, *p* = 0.0039), as well as the DA precursor tyrosine (*H*_(2)_ = 8.526, *p* = 0.014).

These results show that although 20 and 40 mg/L METH induce similar effects, the effect of 40 mg/L METH is stronger and more evident than 20 mg/L METH in most of the assessed endpoints. Therefore, this concentration was selected for the further analysis of the neurotoxic effects (Experiment 2).

### Time-dependent Increase in Methamphetamine Brain Levels

**FIGURE 1:**
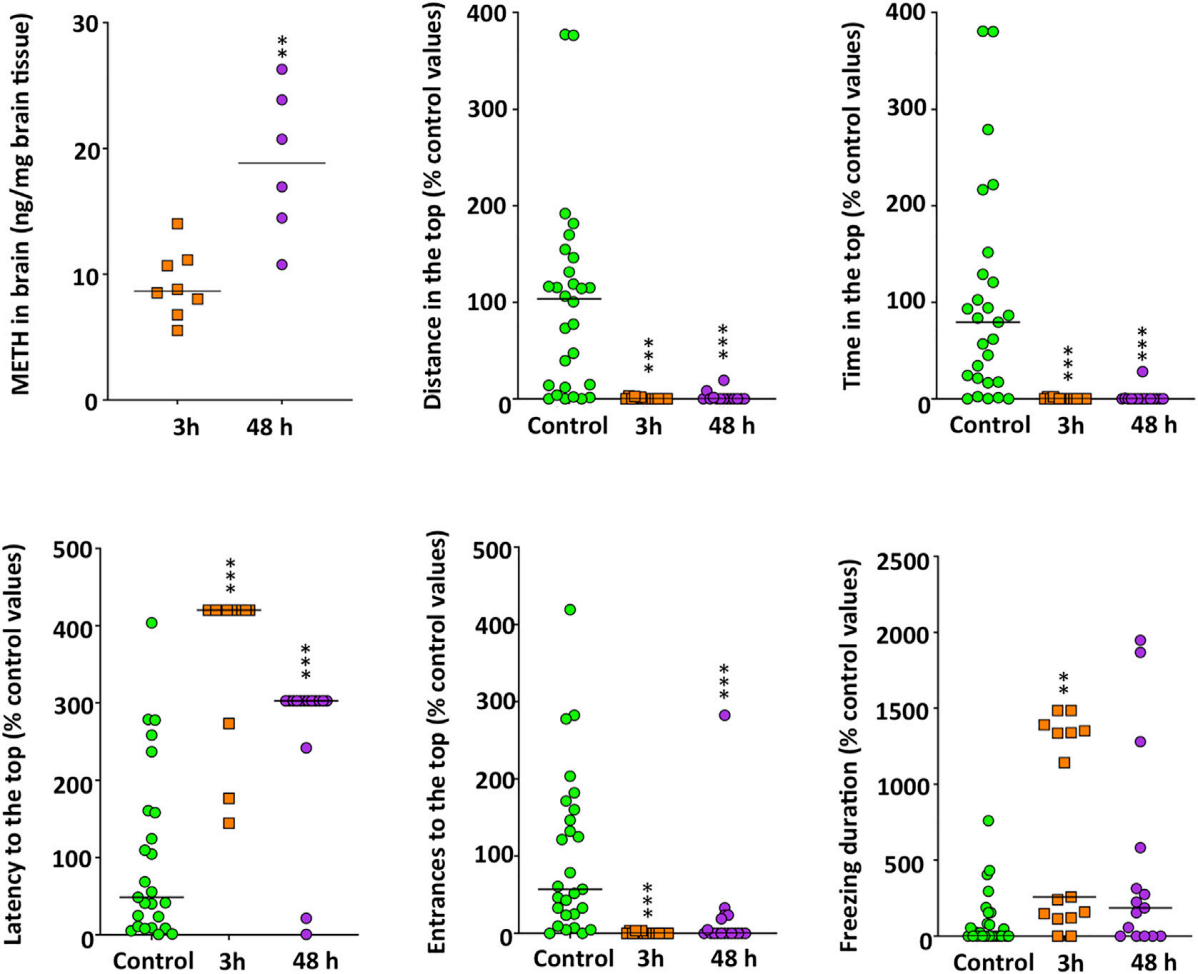
Time-dependent increase in methamphetamine brain levels in adult zebrafish waterborne exposed to 40 mg/L methamphetamine (METH) for 3 and 48 h and anxiety-like behavior, assessed in standard 6-min novel tank test (NTT) of zebrafish exposed to the same concentration and times. Data from each experiment were normalized to the corresponding control values. The combined data is reported as scatter plot with the median (*n* = 6–8 for METH levels in the brain and *n* = 14–15 for the NTT results), **p < 0.01, ***p < 0.001; Student’s *t*-test for METH levels in brain and Kruskal Wallis test with Bonferroni correction for NTT endpoints. Data from two independent experiments.

Concentration of METH in the brain of fish exposed to 40 mg/L METH increased significantly with time (*t*_(12)_ = -4.150, *p* = 0.0013), with levels of 9.19 ± 0.95 and 18.84 ± 2.40 ng/mg brain tissue for 3 and 48 h exposures, respectively (Figure 1).

### Behavioral Effects of Methamphetamine

**FIGURE 2:**
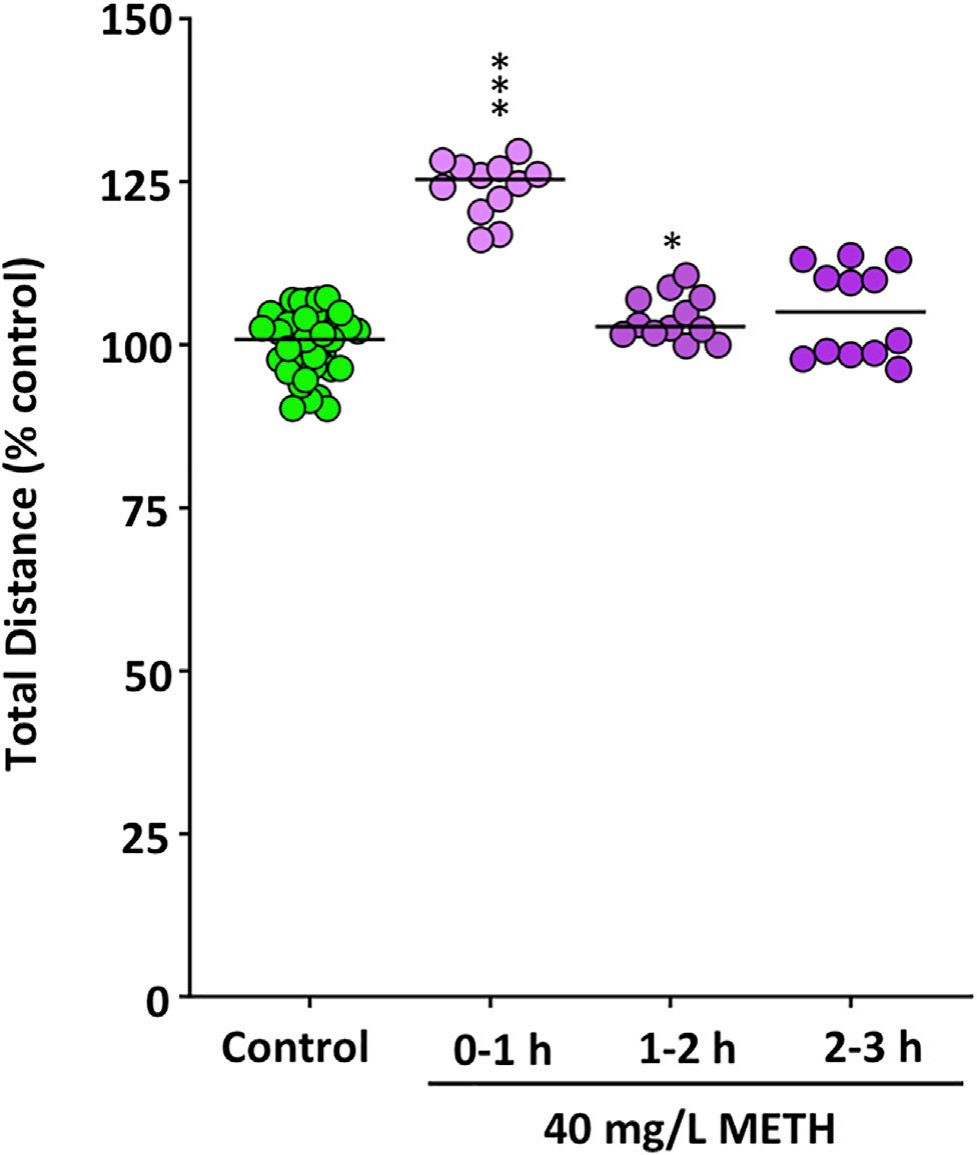
Increased motor activity in fish waterborne exposed to 40 mg/L methamphetamine. The total distance (m) moved during the first hour of the exposure for control fish and exposed fish was measured for each individual fish in shoals of six animals. Data reported as scatter plot with the median (*n* = 12), ***p < 0.001; Student’s *t*-test. Data from two independent experiments.

First of all, the effect of METH on the locomotor activity of the control and METH (40 mg/L)-treated fish was determined per hour during the first 3 h of exposure by measuring both the distance moved and the percentage of time they spent in hypermobility state. As zebrafish is a very social animal, even the short social isolation of testing each fish individually could result in a mild stressful stimulus. Therefore, in order to avoid the potential confounding factor of the social isolation, the locomotor activity was determined in shoals of six fish. As Figure 2 shows, during the first hour of exposure, METH induced hyperactivity, with the exposed fish moving a significant higher distance than controls (*t*_(22)_ = -11.573, *p* = 7.95 × 10^−11^). Moreover, the percentage of time spent in hypermobility state by the exposed fish (*Mdn* = 59.2%) was significantly higher than that of control fish (*Mdn* = 18.6%) [*U*(N_control_ = 12, N_treated_ = 12) = 132.000, z = 3.464, *p* = 0.0002]. During the second hour of exposure, the distance moved by the treated fish was still higher than that of controls, although the significance of the differences was lower (*t*_(22)_ = −2.663, *p* = 0.014), whereas the time spent in hypermobility state by METH-exposed fish (*Mdn* = 57.8%) was also significantly higher than that for control fish (*Mdn* = 21.5%) [*U*(N_control_ = 12, N_treated_ = 12) = 138.000, z = 3.811, *p* = 0.00002]. Finally, although no statistical differences in locomotor activity were found between control and treated fish during the third hour of exposure [*U*(N_control_ = 12, N_treated_ = 12) = 101.000, z = 1.674, *p* = 0.094], hypermobility state in the exposed fish (*Mdn* = 57.5%) was still significantly higher that that in the control fish (*Mdn* = 22.5%) [*U*(N_control_ = 12, N_treated_ = 12) = 122.000, z = 2.887, *p* = 0.0029]. Distance moved in the locomotor assay was also analyzed during the last 6 min of the assay, in order to be able to directly compare locomotor activity in this assay with that in other behavioral 6-min assays also performed after 3 h exposure. In contrast with the results of total distance moved during the 2–3 h period, distance moved by the exposed fish during the last 6-min of the assay (*Mdn* = 977.04 cm) was significantly higher than that for the control (*Mdn* = 900.52 cm) %) [*U*(N_control_ = 12, N_treated_ = 12) = 127.000, z = 3.175, *p* = 0.00086].

In order to better understand the progression of the neurotoxic effects of METH in adult zebrafish, the behavioral effects in animals exposed for 3 and 48 h were analyzed. When the effect of 40 mg/L METH was assessed in the NTT (Figure 1 and Supplementary Figure S6), Kruskal-Wallis analysis showed a significant hypolocomotion (*H*(2) = 37.762, *p* = 6.31 × 10^−9^), a positive geotaxis, characterized by a decrease in the time spent in the top of the experimental tank (*H*_(2)_ = 35.964, *p* = 1.55 × 10^−8^), in the distance moved in this zone (*H*_(2)_ = 32.272, *p* = 9.82 × 10^−8^), and in the number of entrances to this zone (*H*_(2)_ = 36.527, *p* = 1.17 × 10^−8^). METH-treated fish also exhibited a significant increase in the latency to enter for the first time to the top of the tank (*H*_(2)_ = 17.264, *p* = 0.00018). No differences were found in the effect of METH at 3 and 48 h for any of the parameters measured in the NTT when results were adjusted by the Bonferroni correction (*p* > 0.8). However, a significant effect of the exposure time was found for the freezing time (*H*_(2)_ = 13.120, *p* = 0.0014). Whereas the freezing time significantly increased 3 h after exposure (*p* = 0.0012), no differences with the control values were found after 48 h (*p* = 0.16). METH had no effect on erratic movements at any time.

**FIGURE 3:**
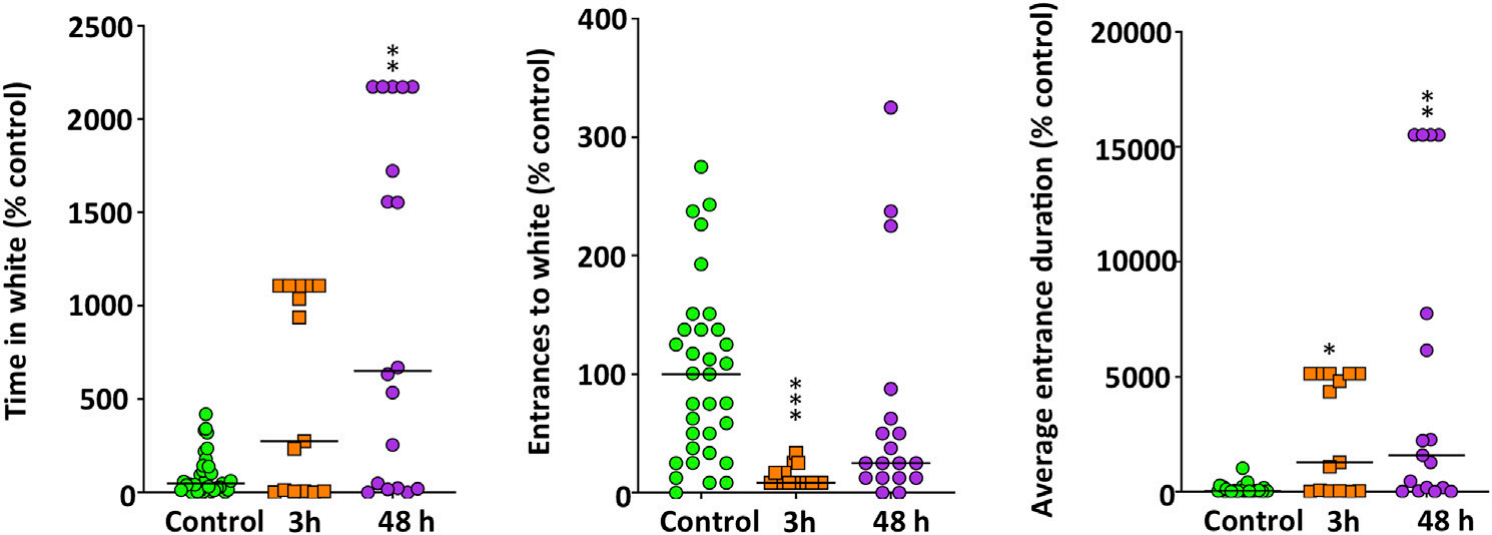
Anxiety-like behavior, assessed in standard 6-min dark-light test (DLT), in adult zebrafish waterborne exposed to 40 mg/L methamphetamine (METH) for 3 and 48 h. Data from each experiment were normalized to the corresponding control values. The combined data is reported as scatter plot with the median (*n* = 15–18), *p < 0.05, **p < 0.01, ***p < 0.001; Kruskal Wallis test with Bonferroni correction. Data from two independent experiments.

The anxiety-like behavior was also assessed by using the DLT (Figure 3). In this experimental paradigm treated fish exhibited a negative scototaxis (*H*_(2)_ = 9.557, *p* = 0.0084), increasing their preference by the white background. Results of the Bonferroni post hoc test indicated that only after 48 h of exposure the treated fish spent significantly more time on the white background (*p* = 0.0071). Moreover, whereas the number of entrances to the white zone significantly decreased in METH-treated fish (*H*_(2)_ = 21.768, *p* = 0.000019), the average duration of each visit to this zone was significantly longer (*H*_(2)_ = 13.478, *p* = 0.0012). Results of the Bonferroni post hoc test did not show significant differences between the two times analyzed for any of the endpoints in the DLT paradigm (*p* > 0.5).

**FIGURE 4:**
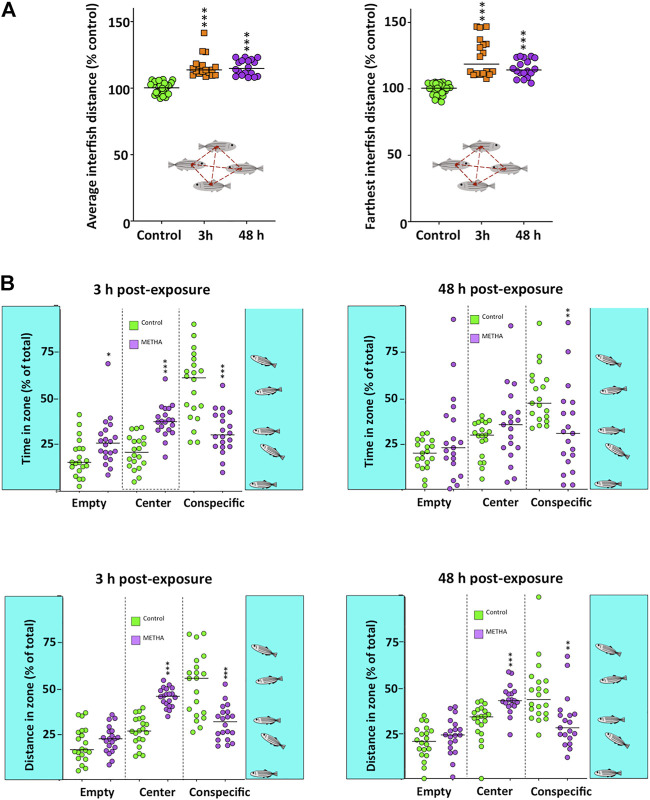
Social behavior in adult zebrafish waterborne exposed to 40 mg/L methamphetamine (METH) for 3 and 48 h. **(A)** Shoaling test results, including the average and the farthest interfish distances. **(B)** Social preference test results, including time and distance of the fish in each of the three virtual zones of the experimental tank: empty, center and conspecific. Data from each experiment were normalized to the corresponding control values. The combined data is reported as scatter plot with the median (*n* = 18 for shoaling test and *n* = 17–20 for social preference test), *p < 0.05, **p < 0.01, ***p < 0.001; one-way ANOVA with Dunnett’s multiple comparison test (Social Preference Test data) or Kruskal Wallis test with Bonferroni correction (Shoaling test data). Data from two independent experiments.

Finally, in order to assess the effects of the acute exposure to METH on the social behavior, two experimental paradigms were used. The first one, the shoaling test (Figure 4A), showed significantly higher average interfish distance (*H*_(2)_ = 53.261, *p* = 2.72 × 10^−12^), farthest interfish distance (*H*_(2)_ = 52.504, *p* = 3.97 × 10^−12^) and nearest interfish distance (*H*_(2)_ = 52.762, *p* = 3.49 × 10^−12^) in the METH-treated fish, a behavioral phenotype consistent with social isolation. No time effect was found by using the Bonferroni post hoc test (*p* > 0.5). Moreover, when the effect of METH on the total distance moved for each fish was measured in this test, a significant effect was found (*H*(_2)_ = 30.612, *p* = 2.52 × 10^−7^). As Supplementary Figure S6 shows, the mobility of the fish in this test was significantly reduced 48 h after exposure (*p* = 1.56 × 10^−7^), but not 3 h after exposure (*p* > 0.5). The second paradigm used for assessing the effects on social behavior was the social preference test (Figure 4B). Results from this paradigm show a significant decrease in both the time that the treated fish spent in the zone closest to the conspecifics (*F*_(2,74)_ = 14.497, *p* = 4.87 × 10^−6^) and the distance moved in this zone (*F*_(2,73)_ = 13.461, *p* = 0.00001). These results are consistent with the social isolation phenotype suggested by the shoaling test results. Results of the Tukey’s Honest Significant Difference (HSD) post hoc test did not show significant differences between the two times analyzed (*p* > 0.5).

### Methamphetamine Decreases Monoaminergic Neurotransmitters

**FIGURE 5:**
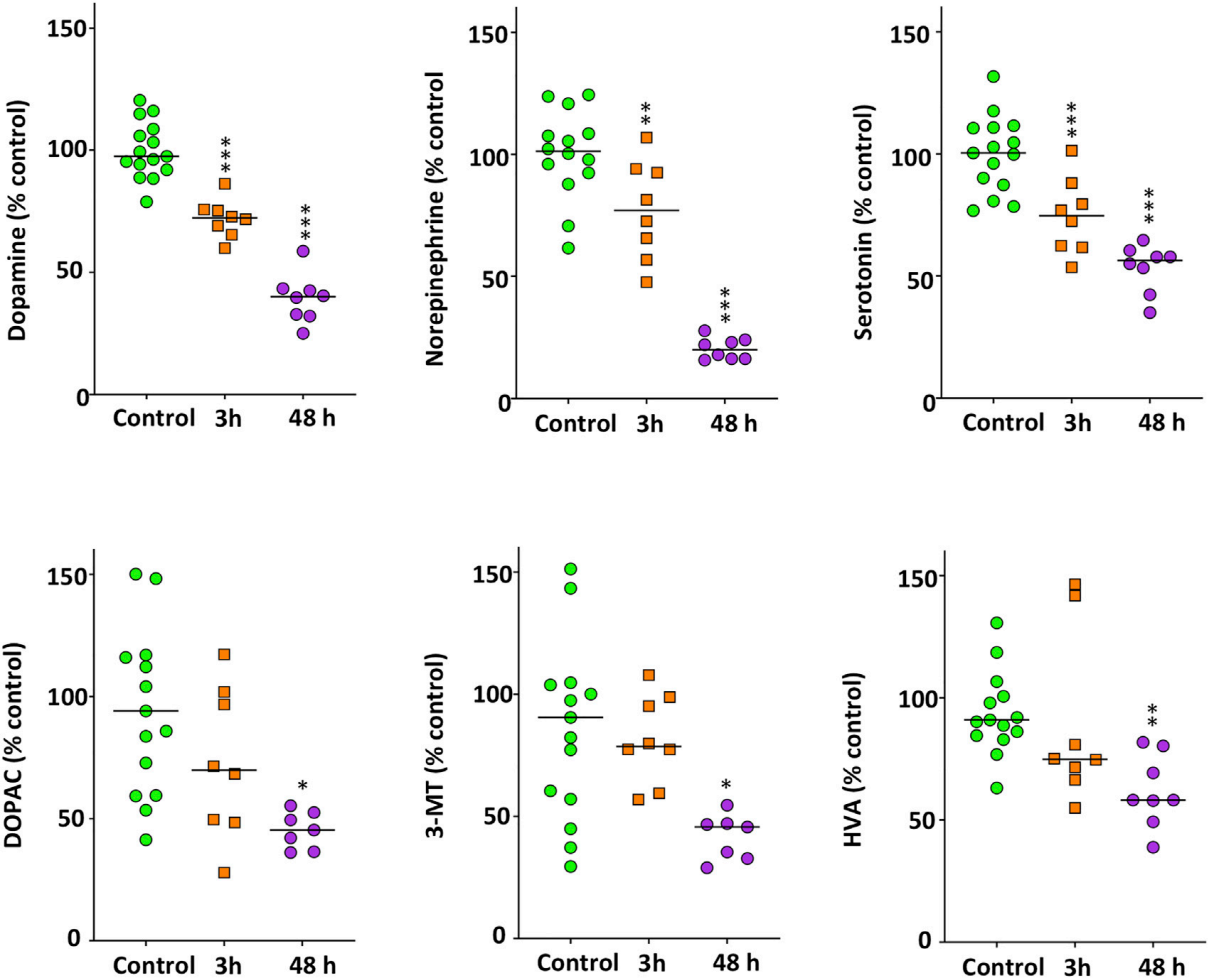
Significant decrease in the monoaminergic neurotransmitters and other neurochemicals in the brain of zebrafish waterborne exposed to 40 mg/L methamphetamine for 3 and 48 h. In each experiment, levels of each neurochemical were normalized to the corresponding control values. For each neurochemical, the combined data is reported as scatter plot with the median (*n* = 7–8 for 3 h exposure and *n* = 15–16 for 48 h exposure), *p < 0.05, **p < 0.01, ***p < 0.001; one-way ANOVA with Dunnett’s multiple comparison test (dopamine, norepinephrine, serotonin) or Kruskal Wallis test with Bonferroni correction (DOPAC, 3-MT, HVA). Data from two independent experiments.

Figure 5 shows the main changes in the monoaminergic neurochemicals profile occurring in the brain of zebrafish exposed for 3 and 48 h to 40 mg/L METH. First of all, a significant decrease was found in DA (*F*_(2,35)_ = 55.503, *p* = 1.39 × 10^−11^), NE (*F*_(2,34)_ = 66.932, *p* = 1.62 × 10^−12^) and 5-HT levels (*F*_(2,35)_ = 33.209, *p* = 8.21 × 10^−9^), the three most relevant monoaminergic neurotransmitters. Results of the Tukey’s HSD test showed that the effect of METH on these neurotransmitters was time-dependent, with the maximum effect after 48 h of exposure (*p* = 0.00016 for DA, *p* = 2.04 × 10^−7^ for NE and *p* = 0.016 for 5-HT). Interestingly, the effect of METH on the dopaminergic system was not restricted to DA, as a significant decrease was also observed in the precursor tyrosine (*H*_(2)_ = 9.621, *p* = 0.008) as well as in the main metabolites of this neurotransmitter, including DOPAC (*H* ^(2)^ = 8.240, *p* = 0.016), 3-MT (*H*_(2)_ = 8.202, *p* = 0.017) and HVA (*H*_(2)_ = 12.378, *p* = 0.002). Although the levels of these neurochemicals exhibited a general trend to decrease with time, results of the Bonferroni post hoc test only showed significant differences for those animals exposed for 48 h respect to the control fish (*p* = 0.006 for tyrosine, *p* = 0.016 for DOPAC, *p* = 0.013 for 3-MT and *p* = 0.001 for HVA). Regarding 5-HIAA, the degradation product of 5-HT, no differences with the control were found (*p* > 0.05).

### Effects of Methamphetamine on Gene Expression

**FIGURE 6:**
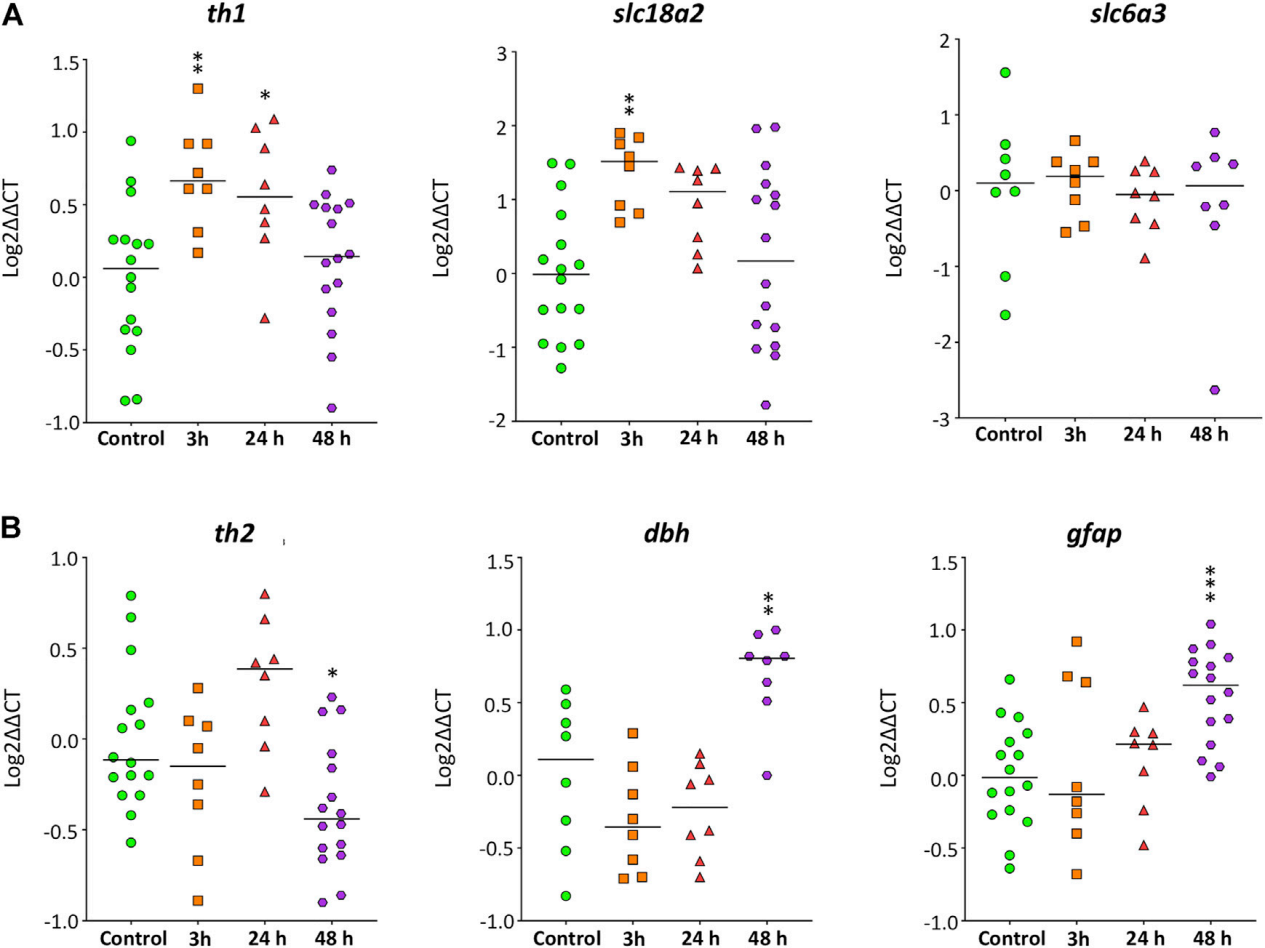
Time-course of the expression of genes involved in the synthesis and transport of catecholamines and neuroinflammation in zebrafish brain after waterborne exposure to 40 mg/l methamphetamine. **(A)** Expression of *th1* and *slc18a2,* but nor *slc6a3* exhibits an early response to methamphetamine. **(B)** Expression of *th2*, *dbh* and *gfap* exhibits a late response. Data reported as scatter plot with the median. *p < 0.05, **p < 0.01; one-way ANOVA with Dunnett’s multiple comparison test (*th1*, *th2*, *dbh*, *slc18a2*) or Kruskal Wallis test with Bonferroni correction (*slc6a3*, *gfap*). Data from two to three independent experiments (*n* = 8–16 for *th1*, *th2* and *gfap* and *n* = 8 for *slc18a2*, *dbh* and *slc6a3*).

First, the expression of five genes involved in the synthesis (*th1*, *th2*, *dbh*) and transport (*slc6a3, slc18a2*) of catecholaminergic neurotransmitters were analyzed in the brain of zebrafish control and exposed to 40 mg/L METH for 3, 24, and 48 h. Figure 6 shows a significant effect of exposure time to METH on the expression levels for *th1* (*F*_(3,44)_ = 5.660, *p* = 0.002), *th2* (*F*_(3,44)_ = 6.684, *p* = 0.001), *dbh* (*F*_(3,28)_ = 11.180, *p* = 5.36 × 10^−5^), and *slc18a2* (*F*_(3,44)_ = 4.990, *p* = 0.005), but not for *slc6a3* (*H*_(3)_ = 4.838, *p* = 0.184). *th1* and *slc18a2* exhibited an early response to METH, increasing their levels significantly (*p* = 0.004) after only 3 h of exposure. The levels of *th1* remained increased at 24 h (*p* = 0.022), returning to the control values 48 h after exposure (*p* = 0.844). In contrast with *th1* and *slc18a2*, the expression of *th2* and *dbh* was altered by METH at the end of the exposure. Whereas the levels of *th2* significantly decreased (*p* = 0.019) at 48 h after exposure, the levels of *dbh* increased (*p* = 0.004).

The expression of *gfap* was also analyzed (Figure 6), as this gene is a classical marker of neuroinflammation. The exposure time to METH had a significant effect on *gfap* expression (*F*_(3,44)_ = 6.534, *p* = 0.001), with the levels of this transcript strongly increased after 48 h of exposure (*p* = 0.0003).

### Effects of Methamphetamine on Lipid Peroxidation

No differences in lipid peroxidation levels in the brain were found between control and fish exposed for 48 h to 40 mg/L METH [15.78 ± 0.95 vs 13.49 ± 0.73 pmol MDA/mg ww for control (*n* = 10) and METH-exposed (*n* = 9) fish, respectively; *t*_(17)_ = 1.216, *p* = 0.066; Data from three independent experiments].

## Discussion

Zebrafish has become a relevant vertebrate model species in neurophenotyping neuroactive compounds and abuse drugs (Neelkantan et al., 2013; Kyzar and Kalueff, 2016). However, although a few recent studies have analyzed some of the addictive effects of METH in adult zebrafish (Mi et al., 2016; Zhu et al., 2017), information on the neurotoxic effects of the acute exposure to this stimulant drug is currently missing. One interesting potential advantage of using zebrafish for modeling methamphetamine neurotoxicity is that, as this animal is a vertebrate poikilotherm, it should be possible to perform mechanistic studies without the confounding factor of hyperthermia (Xie et al., 2000). In this study we have continuously exposed adult zebrafish to a METH dose of 40 mg/L for 48 h, and the effects on the brain monoaminergic profile, locomotor, anxiety-like and social behaviors as well as on the expression of key genes of the catecholaminergic system have been determined.

METH levels determined in the brain of chronic human consumers are in the range 0.24–56.6 ng/mg brain tissue, and the distribution among the different brain areas seems quite homogeneous (Wilson et al., 1996; Kalasinsky et al., 2001; Moszczynska et al., 2004). Similarly, in rodents, METH levels determined in different brain areas after a binge of METH (5 injections of 12.5 mg/kg every 4 h) were about 8 ng/mg brain tissue (Danaceau et al., 2007). However, other authors reported higher METH levels (≈30 ng/mg brain tissue) in the whole brain of rats after similar dosing schedule (four to five injections of 11.5–15 mg/kg every 2–6 h interval) (Alburges et al., 1990; Gygi et al., 1996) (Alburger et al., 1990; Gygi et al., 1996). In our study, the average METH concentration in the brain of fish after 48 h of exposure to 40 mg/L METH was 18.8 ng/mg brain tissue, which is in the range of the described in human and rodent models. Peak plasma concentration of METH has been reported to occur as early as 3–5 h after dosing (Kalasinsky et al., 2001), which should be reflected in an early increase in brain levels of this drug. In this study, the average brain levels of METH 3 h after exposure were 9.19 ng/mg brain tissue suggesting a rapid increase in METH plasmatic levels, explaining some of the behavioral and neurochemical changes found at this time.

In mammalian models, after an initial release of DA and 5-HT, acute high doses of METH causes damage to dopaminergic and serotonergic axon terminals in the brain. As a result, a decrease in the DA and 5-HT in the brain is commonly used as a neurochemical marker of METH neurotoxicity (Yu et al., 2015). Similarly, a concentration- and time-dependent decrease in the levels of DA, NE and 5-HT were found in the brain of the treated fish. Moreover, the levels of the DA metabolites DOPAC, 3-MT and HVA also decreased after 48 h exposure to 40 mg/L METH, an effect probably related with the decreased DA levels, although it is not possible to discard the potential involvement of the monoamineoxidase (MAO) activity inhibition (Egashira and Yamanaka, 1993; Xie et al., 2000; Escubedo et al., 2011).

The observed changes in the neurochemical profile in the brain of the treated fish are consistent with the observed changes in behavior. First of all, the fact that fish exhibited a significant hyperactivity during the first hour of exposure to 40 mg/L METH, and then the activity returned to the control values strongly suggest that after the initial release of DA, the levels of this neurotransmitter decline very fast. However, the similar distance moved by the control and treated fish in the locomotor activity assay by the end of the 3 h exposure period contrast with the highly significant decrease in mobility observed in the exposed fish during the 6 min of the NTT performed immediately after the 3 h exposure (Supplementary Figure S3). The important differences in the design of both behavioral assays might by behind the differences in the results. In the locomotor activity assay shoals of six fish are placed into experimental tanks and video-recorded for 180 min. Under these conditions, fish do not have stressful stimuli such as handling induced stress, novelty or social isolation. In the NTT, however, fish are transferred from the exposure tank (handling induced stress) to the observation tank (stress induced by a novel environment) individually (stress associate to social isolation). Interestingly, in the shoaling test, an assay where the fish is exposed to the stress associated with handling and the novel environment, but not to social isolation, no effects on mobility were observed 3 h after exposure, a result consistent with that obtained in the locomotor assay, another test performed on shoals. This result suggests that the interaction of METH with social isolation, a stressful stimulus in highly social species like zebrafish, is the behind of the observed differences in the mobility between the locomotor and NTT assays. In rodents and zebrafish, stressors have been shown to potentiate the effects of drugs on locomotor activity (Arias et al., 2010; Tran et al., 2016), but the effect of the interactions between neuroactive drugs and stressors on locomotor activity is generally difficult to interpret (Tran and Gerlai, 2016).

In this study METH-treated fish exhibited a significant effect on the anxiety-like behavior, spending more time on the bottom of the tank (positive geotaxis) and increasing the preference for the white background (negative scototaxis) compared to the corresponding controls. Positive geotaxis, with decreased time and distance in the top of the tank, has been previously reported in adult zebrafish acutely water-exposed to 5–10 mg/L d-amphetamine (Kyzar et al., 2013) and 2 mg/L METH (Mi et al., 2016).

Anxiety-like behaviors have been positively correlated with brain serotonin levels in the novel tank test, whereas this correlation is negative in the light/dark test (Maximino et al., 2016). For instance, when 5-HT levels were decreased with para-chlorophenylalanine (PCPA) in the brain of adult zebrafish, animals exhibited positive geotaxis (increased time on the bottom) and negative scototaxis (decreased time on the dark background) (Maximino et al., 2013). These results are similar to the situation in zebrafish exposed to METH in the present study, with decreased brain levels of serotonin, positive geotaxis and negative scototaxis, strongly suggesting that the effects of METH on the anxiety-like behavior are mediated by the decrease in brain 5-HT.

In this study, METH-treated fish also exhibited impaired social behavior in both the shoaling test and the social preference test. This result is consistent with a number of studies with rats and monkeys, where the acute and chronic exposure to METH results in social withdrawal (reviewed in Homer et al., 2008). In humans, changes in social behavior also associated with METH abuse have been explained by impairments in social-cognitive function (Homer et al., 2008).

Expression of *slc18a2*, the gene encoding the vesicular monoamine transporter 2 (VMAT2), was upregulated after only 3 h of exposure, a finding suggesting a rapid inhibition of VMAT2 activity in the exposed fish. VMAT2 is one of the molecular targets of amphetamine and its derivatives. It’s well known that the disruption of VMAT activity in mammalian models by genetic knockdown or by chemical inhibitors results in a significant decrease in brain monoaminergic neurotransmitters. Interestingly, the brain of *Vmat2* heterzygous mutant zebrafish also showed decreased levels of the monoaminergic neurotransmitters DA, NE and 5-HT, as well as some of their metabolites. Moreover, these mutants displayed altered anxiety-like behavior, with positive geotaxis and negative scototaxis (Wang et al., 2016). Although VMAT2 activity has not been determined in this study, the similarity between the neurochemical and behavioral phenotypes of the *Vmat2* mutants and the METH-treated fish suggests that VMAT inhibition plays also an essential role in the neurotoxic effects of METH in adult zebrafish.

Information about the effects on gene expression of METH and other amphetamine derivatives in zebrafish brain is very scarce. An early and transient upregulation *th1*, gene encoding the rate-limiting enzyme in the synthesis of catecholamines, has been found in fish acutely exposed to METH in this study. Acute exposure to METH has also been reported to induce a transient early overexpression of this gene in specific nuclei of rodent brain (Shishido et al., 1997; Ferrucci et al., 2007; Braun et al., 2011). Moreover, in the only study that to our knowledge has analyzed the effect of d-amphetamine on tyrosine hydroxylase transcription in fish (*Carassius auratus*), an early up-regulation was also found in the expression levels of this gene at the telencephalon (Volkoff, 2013). Interestingly, the tyrosine hydroxylase gene regulated by METH in this fish species is homologous to zebrafish *th1* (Supplementary Figure S7), the gene exhibiting an early upregulation by METH in our study. Treatment with high doses of METH induces a significant increase in PKCδ expression in the striatum, an effect resulting in a significant inhibition of TH activity by phosphorylation at Ser40 (Dunkley et al., 2004). The observed upregulation in *th* mRNA expression after high doses of METH might be a mechanism trying to counteract this inhibition in TH activity.

In contrast with the early increase in the expression of *th1* found in the brain of METH-exposed fish in this study, *th2* was found to be significant downregulated. Interestingly, *th2*, but not *th1*, was reduced in the telencephalon of zebrafish exposed to cocaine (Darland et al., 2012), an illicit drug also targeting dopaminergic system. Moreover, mild oxidative stress has been reported to induce a clear decrease in *th2*, but not in *th1* expression in zebrafish (Priyadarshini et al., 2013). Whereas we have not been able to detect oxidative stress in the whole brain of the exposed fish, it is not possible to discard the generation of oxidative stress at specific areas enriched in dopaminergic terminals. Therefore, the downregulation of *th2* found in the brain after 48 h of exposure to METH is probably a much more sensitive marker of oxidative stress at specific brain areas (Priyadarshini et al., 2013) than the LPO levels on the whole brain.

Whereas a persistent loss of DA transporter (DAT) in the cortex and caudate-putamen is one of the neurodegenerative changes observed in the brain of human addicts (Cadet and Krasnova, 2009), no differences in the expression on *slc6a3*, the gene encoding DAT, have been found in the brain of the exposed fish at any of the selected times. However, effects of METH at the protein and transcript levels are often different. For instance, METH challenges induced a significant decrease in DAT (protein) levels in the midbrain of rats, with no effect on *slc6a3* (transcript) levels (Krasnova et al., 2011). No differences in the expression of *slc6a3* were either found in the brain of METH conditioned preference place rats (Hensleigh and Pritchard, 2014).

Glial fibrillary acid protein (GFAP) is commonly used as an index of astroglial response to neuronal injury. In rodent models, METH exposure led to an up-regulation of GFAP, both at transcript and protein levels (Sriram et al., 2002, 2006; Shen et al., 2011; Robson et al., 2014). Therefore, the up-regulation in *gfap* expression observed in the brain of zebrafish after 48 h exposure to 40 mg/L METH is consistent with the effect of this drug on mammalian models.

Despite the early upregulation of *th1* and *slc18a2* expression observed in the brain of the METH-exposed fish, DA levels significantly decreased between the 3 and 48 h of exposure to 40 mg/L METH, a result suggesting a quick turnover rate for the recently synthesized TH and VMAT2 proteins induced by METH. However, other potential explanations to the absence of recovery of DA levels include a specific effect of METH on mRNA translation, post-translational modifications (Zhu et al., 2016) of TH and VMAT2 or a high rate of impairment of dopaminergic terminals by the end of the exposure period, as suggested by the significant increase in *gfap* expression, counteracting any potential increase in DA synthesis resulting from the early increase in *th1* expression.

Finally, an unexpected finding in this study has been that the brain of the exposed fish had levels of lipid peroxidation similar to those of controls. METH neurotoxicity has been reported to depend on the formation of DA quinones and superoxide radicals within the nerve terminals. Moreover, it is known that METH decreases the levels of reduced glutathione (GSH) and saturates the capacity of the antioxidant defense system, increasing lipid peroxidation in the dopaminergic neurons (Cadet and Krasnova, 2009). The most suitable explanation to the absence of evidences of oxidative stress in this study is that oxidative stress is produced at the dopaminergic terminals, but the lipid peroxidation analysis has been performed on the whole brain. In this context, the increase in oxidative stress at very restricted locations would be diluted and non-detected when the whole brain is analyzed, as in our study. On the other hand, a number of studies support that the adult zebrafish brain exhibits an antioxidant defense system with high capacity. For instance, no lipid peroxidation was recently found in a zebrafish model for acute acrylamide neurotoxicity, despite the brain exhibited a total depletion of glutathione levels and a severely impaired thioredoxin system (Raldúa et al., 2020). No lipid peroxidation was either found in the brain of zebrafish treated with paraquat at concentrations inducing 30–40% lethality, although the exposure to this herbicide resulted in a decrease in the superoxide dismutase (SOD) activity, catalase activity and GSH content (Anand et al., 2021). However, in our study, only lipid peroxidation has been determined as indicator of oxidative stress, and the evaluation of the effects on the different components of the antioxidant defense system were out of the scope of the study. Moreover, another explanation for the lack of oxidative stress evidenced in our study could be related to the non-hyperthermic response in adult zebrafish since there are several mechanisms by which hyperthermia may enhance METH-induced neurotoxicity to DA terminals. It is known that METH-induced hyperthermia directly increases ROS formation in rats and mice striatum and cause an increase in ROS-induced gene expression (Cadet and Brannock, 1997; Imam et al., 2001; Krasnova et al., 2001; Cadet et al., 2007); for review see (Bowyer and Hanig, 2014). In fact, hyperthermia alone produces equivalent increases in genes up-regulated by ROS in some brain regions (Bowyer et al., 2013).

To sum up, data presented in this manuscript support the suitability of the adult zebrafish model for studying the neurotoxic effects of a binge-like methamphetamine exposure without to potential confounding factor of hyperthermia. Interestingly, the absence of oxidative stress in the phenotype of the brain of METH-treated fish has allowed to identify some relevant components of METH neurotoxicity that are oxidative stress-independent, such as the observed behavioral effects, the reduction of monoamines content, the effects on *th1, th2 and slc18a2* expression, as well as an astroglial response. However, further studies are needed in order to evaluate the presence of oxidative stress in the dopaminergic terminals and the different components of the antioxidant defense system of the exposed fish.

## Data Availability

The raw data supporting the conclusions of this article will be made available by the authors, without undue reservation.
