# Supplementary material for: A Zebrafish Model of Neurotoxicity by Binge-Like Methamphetamine Exposure
Source: Front Pharmacol. 2021 Nov 22;12:770319. doi: 10.3389/fphar.2021.770319 (PMC8646101; doi:10.3389/fphar.2021.770319)
Supplement: Supplementary file 1 [file DataSheet1.PDF]

Supplementary Information

for

A zebrafish model of neurotoxicity by binge-like methamphetamine exposure

Juliette Bedrossiantz , Marina Bellot, Pol Dominguez-García, Melissa Faria, Eva Prats, Cristian Gómez-Canela, Raul López-Arnau, Elena Escubedo, Demetrio Raldúa*.

Email: drpqam@cid.csic.es

This PDF file includes:

Supplementary Methods

Figs. S1 to S7

Tables S1 to S2

Supplementary Methods

Behavioral testing

All tests were performed in an isolated behavior room at 27–28°C. Animals (≈50:50 male: female ratio) were brought to the behavioral room one hour before testing began, to acclimate to this new environment, and then, behavioral testing was conducted between 10:00 and 17:00 h. All fish used in this study were experimentally naïve and all the testing was performed in a blind manner, with observers unaware of the experimental group. Whereas in Experiment 1 only the novel tank test (NTT) was performed, in Experiment 2 four different behavioural tests were analysed. For the later experiment, we conducted a test battery approach, which is more suitable for behavioral and ethical testing, as it reduces the number of animals needed for this study (3Rs principle - reduction). As no test battery effect has been reported in batteries of NTT and the Dark-Light Test (DLT) when they are performed one after the other (Kysil et al., 2017), LDT was performed after NTT in Experiment 2. Moreover, a test battery approach was also used with the social tests, performing the Social Preference Test (SPT) after the Shoaling Test. The average interval between the two tests of each battery was 2 h, and during this period animals were housed individually, but maintaining visual contact. In order to avoid any potential tank effect, the experimental group assigned to each tank was switched between trials. Fish exposed to the compound were previously washed several times in clean water to avoid contaminating the behavioral tank and the control animals.

Novel Tank Test (NTT)

The novel tank test (NTT), used to assess basal locomotor activity, exploratory behavior, geotaxis and freezing movements of fish, was performed using an experimental setup allowing monitoring and recording 3 fish simultaneously. The NTT was performed in three experimental tanks (20 cm length, 20 cm width, 25 cm height) containing 7 L (20 cm height) fish water at 28 °C. LED backlight illumination (GP-G2, Quirumed, Spain) located behind the tank provided uniform illumination for video-recording. Control and METH-exposed fish were tested in the standard 6-min NTT. Each trial was video-recorded (AVI format, 30 fps) with the uEye Cockpit software (version 4.90; Imaging Development Systems, Germany) controlling a GigE camera (UI-5240CP-NIR-GL, Imaging

Development Systems, Germany) mounted in front of the experimental tank. Once the recording was completed, the videos were analysed by Ethovision XT 13.0 (Noldus, Wageningen, the Netherlands). First of all, the front of the tank was divided into two equal virtual zones, top and bottom.

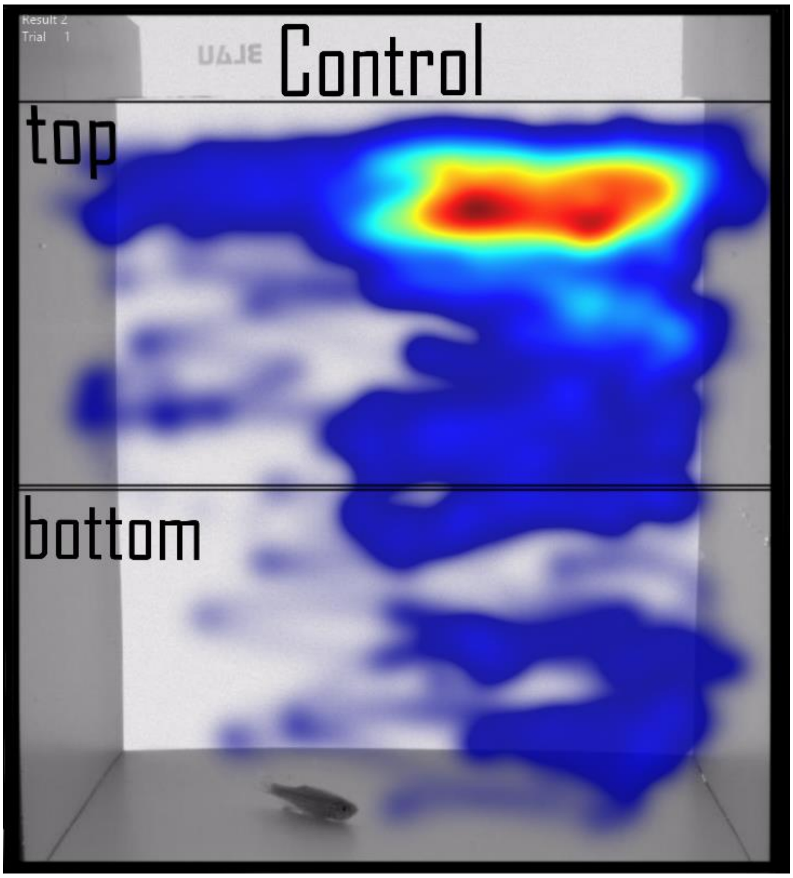

Then, the total distance travelled (cm), distance travelled in the top and in the bottom (cm), time spent in the top (s), latency to top (s), and entrances to top were determined. Moreover, acceleration state (frequency and duration (s) for high and low accelerations) mobility states (s) as hypermobility (high mobility criteria: superior to 70% mobility during a minimum continuous time superior to 150 frames; 5s) and complete immobility (immobile criteria: inferior to 3% mobility during a minimum continuous time superior to 150 frames; 5s) as well as number and duration (s) of freezing bouts were calculated by the software, in order to evaluate the occurrence of complex behaviours as darting/erratic, bursts of rapid movements, and freezing movements in between, reflecting the anxiety state of the animals.

Dark and Light Test (DLT)

The dark-light test (DLT), used to determine scototaxis and thigmotaxis of the fish, was performed using an experimental setup allowing monitoring and recording 2 fish simultaneously. The DLT was performed in two experimental tanks (38 cm length, 24 cm width, 28 cm height) with one horizontal half made of white acrylic and the other half made of black acrylic. Each experimental tank contained 9 L (10 cm height) of fish water at 28 °C. Two anti-flicker LED tubes (TUT8-ST28-NFL; AS de LED, Valencia, Spain) mounted on both sides of the test tanks provided uniform illumination for video-recording. Light intensity in the dark and light zones, measured with an Iso-Tech-1332 digital illuminance meter (Iso-Tech LTD, England), was in the range of 250–300 lux. The first 6 min of the trial were video-recorded (AVI format, 30 fps) with the uEye Cockpit software (version 4.90; IDS GmbH, Germany) controlling the GigE cameras (UI-5240CP-NIR-GL, IDS GmbH, Germany) placed on top of the white part of the testing tanks. The recorded videos were analysed by Ethovision XT 13.0.

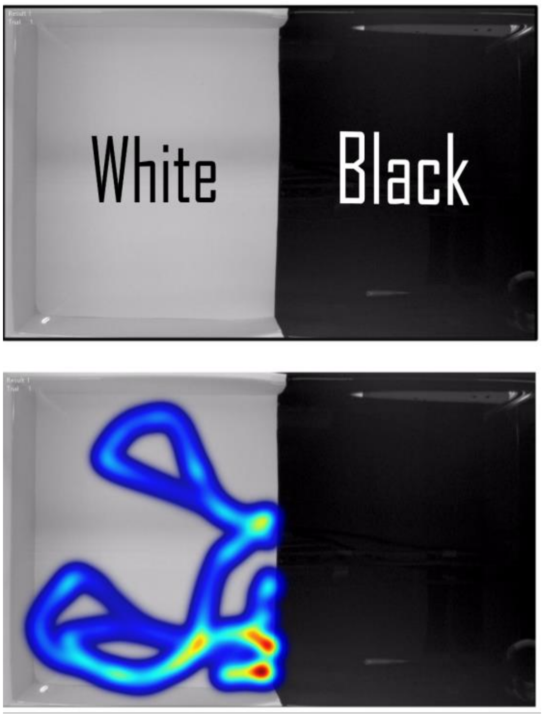

Then, the time spent in the black and white chambers (s), the latency to enter to the white chamber (s) as well as the frequency to enter to the white chamber were determined.

Shoaling Test

The shoaling test provides information on both social and anxiety-like behaviors. Two independent trials were performed, with a shoal size of 9 fish per experimental group in each (n=18). Groups of 9 zebrafish from the control and methamphetamine groups were video-recorded for 6 min in our novel tank, and analysed using Ethovision XT 13.0 social interaction module (SIM). This automated method allows us to detect and track each fish in the group and measure inter-fish distances continuously in order to assess the shoal density or the shoaling tendency of each individual. Typical shoaling behaviour parameters were used, including the average distance between fish (cm), the average distance between the nearest neighbour (cm) and the average distance between the farthest neighbour. The information provided by the software is the inter-fish distance for each fish. This parameter measure the distance between two individuals determined from the central point of each fish. From these data, average inter-fish distance was calculated by averaging interfish distances between all members of the shoal, whereas the farthest and nearest interfish distance for each fish is directly extracted from the interfish distance dataset. In addition to these shoaling specific endpoints, the total distance moved (cm) for each fish of the shoal was measured after 3 h of exposure.

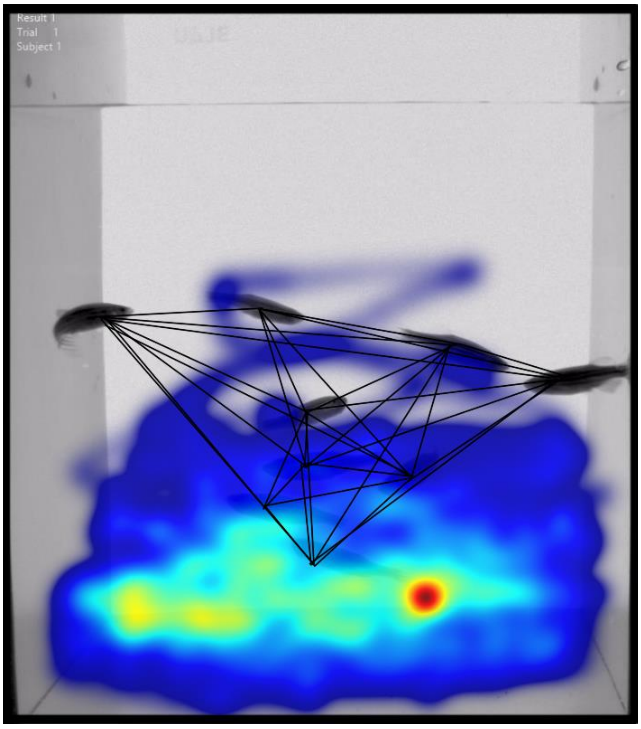

Additional information on the Social Interaction Module of Ethovision can be found at Green et al. (2012).

Supplementary Figures

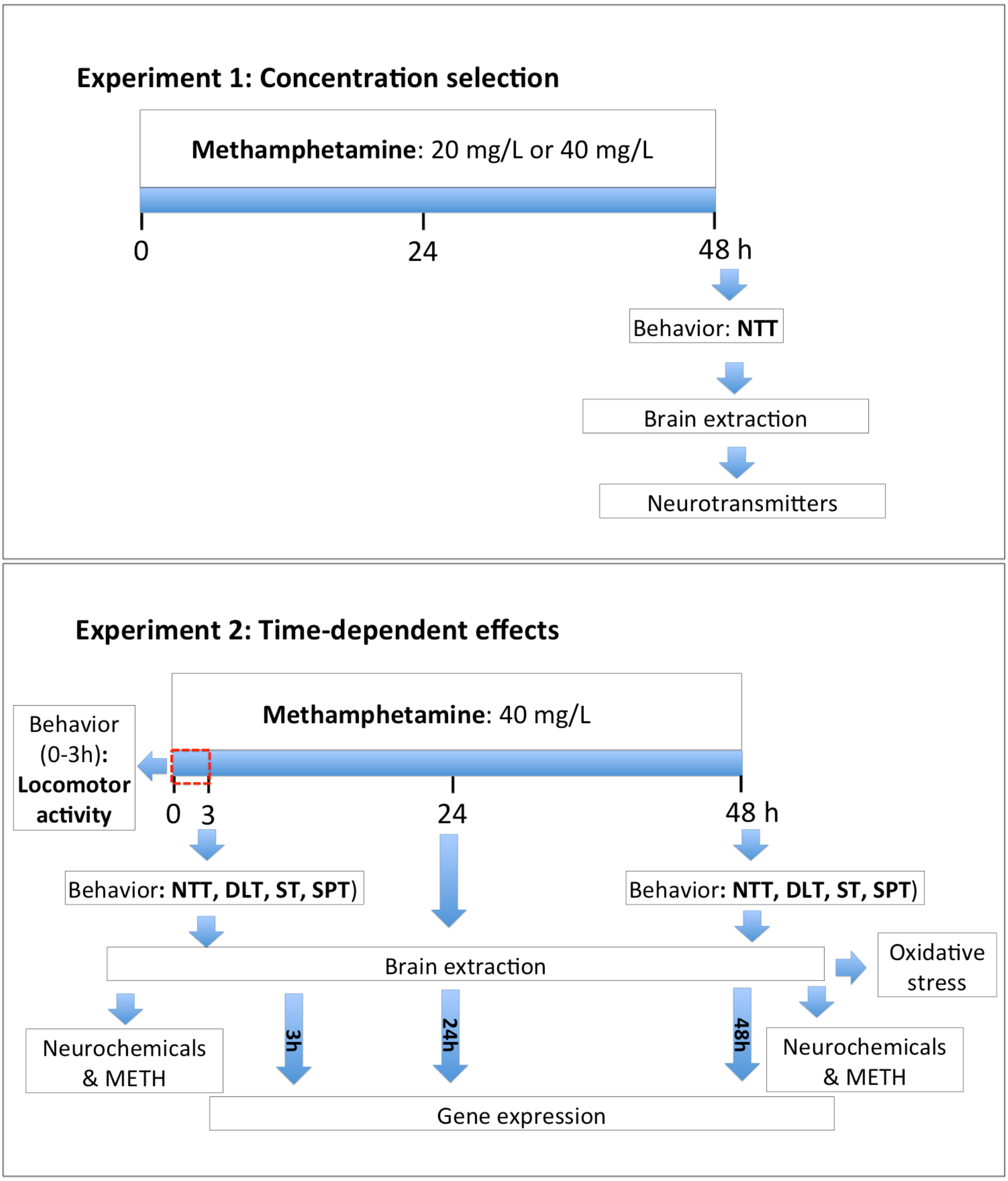

Supplementary Figure S1. Experimental Design of Experiments 1 and 2. NTT: novel tank test; DLT: Dark-light test; ST: shoaling test; SPT: Social Preference Test

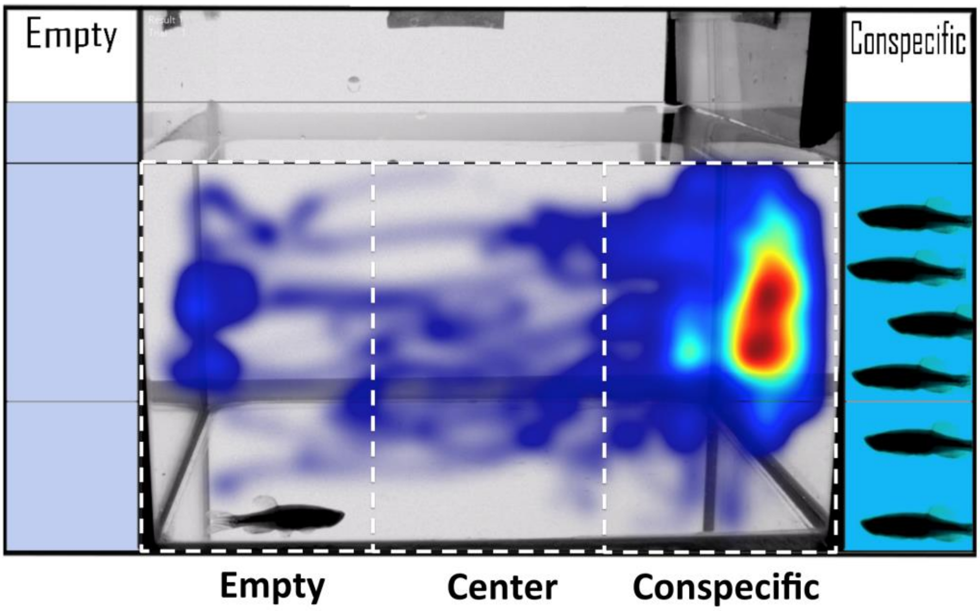

Supplementary Figure S2. The Social Preference Test Tank has a shoal of fish on one side, and an empty tank in the other side. During the video analysis with Ethovision XT 13.0, the tank is divided in three virtual zones: empty/ center/ conspecific.

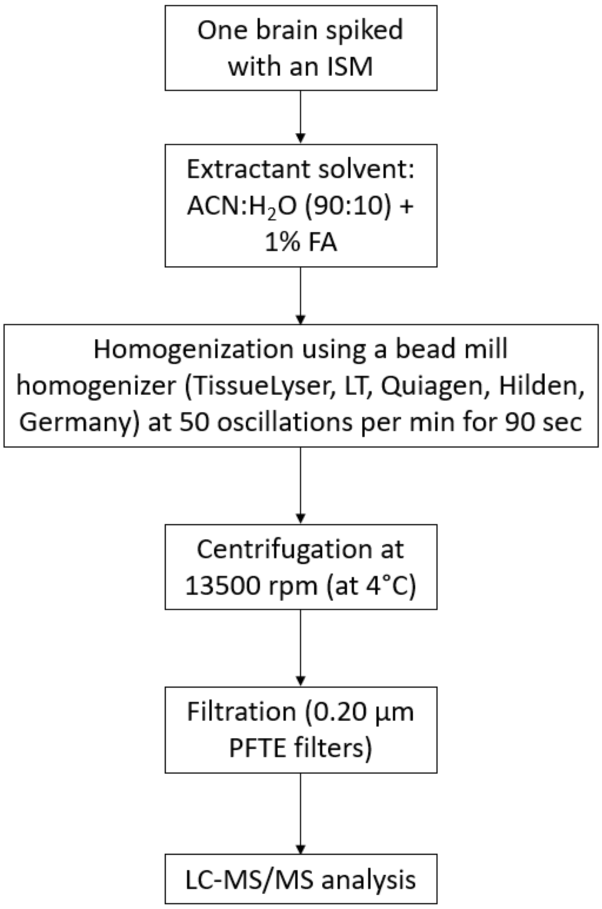

Supplementary Figure S3. Flow chart for methodology used for the extraction of monoaminergic neurochemicals from the zebrafish brains.

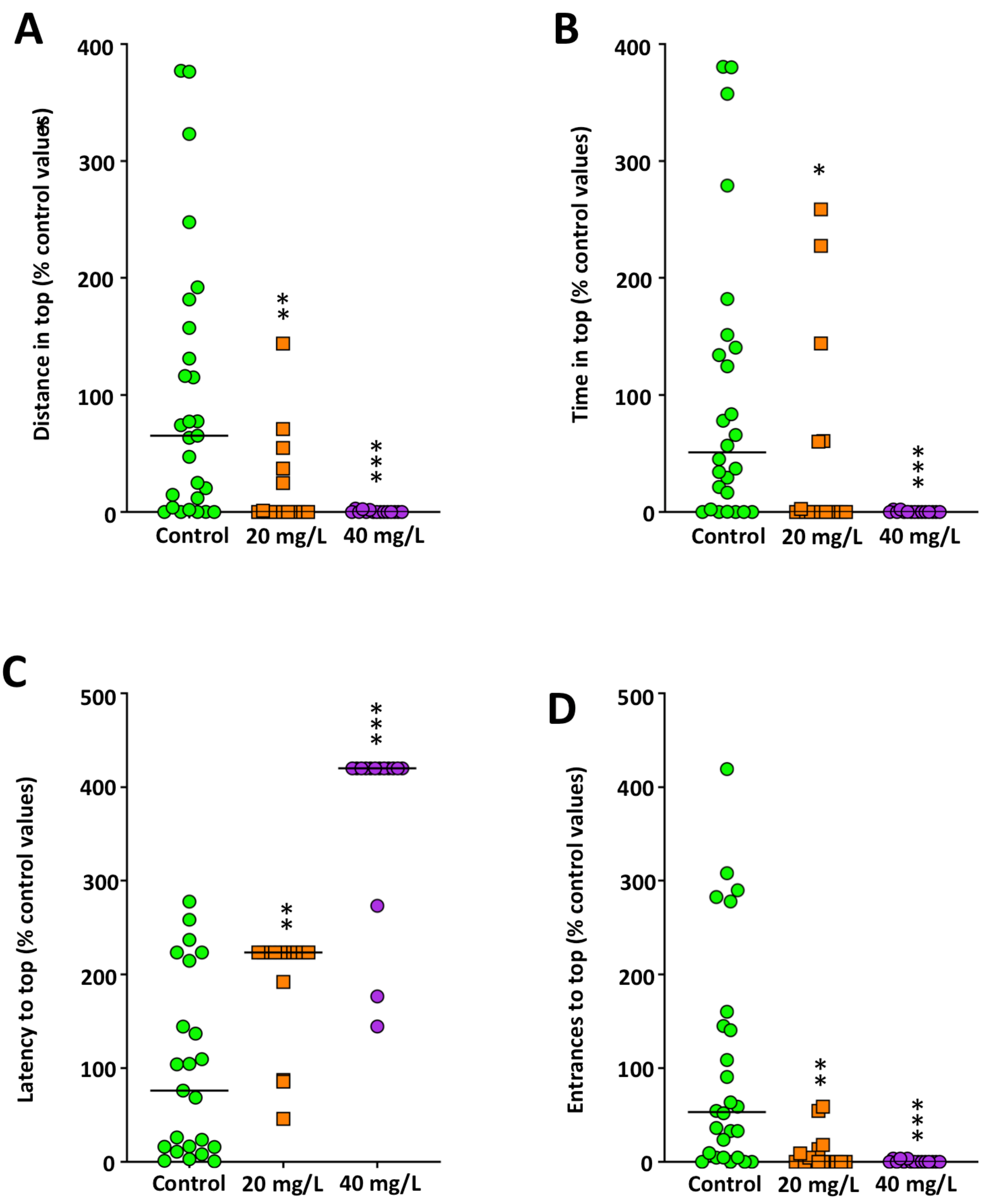

Supplementary Figure S4. Anxiety-like behavior, assessed in standard 6-min novel tank test (NTT), in adult zebrafish waterborne exposed to 20 and 40 mg/L methamphetamine for 48h. (A) Distance moved at the top of the tank; (B) Time spent at the top of the tank; (C) Latency to enter for the first time to the top of the tank; (D) Number of entrances to the top of the tank. Data reported as scatter plot with the median (n = 15-27), *p < 0.05, **p < 0.01, ***p < 0.001; Kruskal Wallis test with Bonferroni correction. Data from 2 independent experiments.

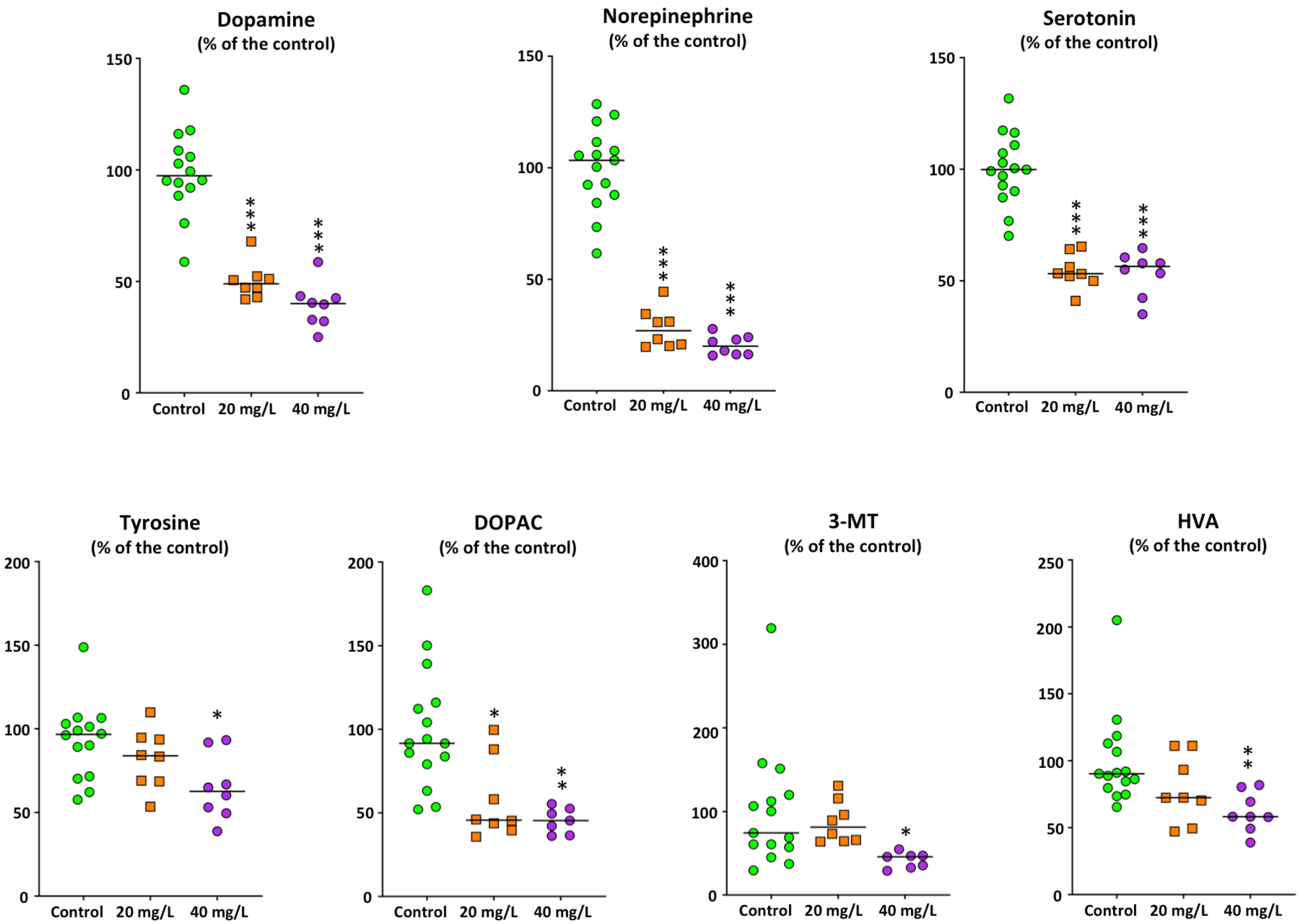
Dopamine
(% of the control)
Norepinephrine
(% of the control)
Serotonin
(% of the control)
Tyrosine
(% of the control)
DOPAC
(% of the control)
3-MT
(% of the control)
HVA
(% of the control)
Control
20 mg/L
40 mg/L

Supplementary Figure S5. Effect of 48h exposure to 20 and 40 mg/L METH on the monoaminergic profile in the zebrafish brain. Concentration of each neurochemical is reported as % of the control values. Data reported as scatter plot with the median (n = 7-15). *$p < 0.05$, **$p < 0.01$, ***$p < 0.001$; one-way ANOVA with Dunnett's multiple comparison test (dopamine, norepinephrine and serotonin) or Kruskal Wallis test with Bonferroni correction (tyrosine, DOPAC, 3-MT and HVA).

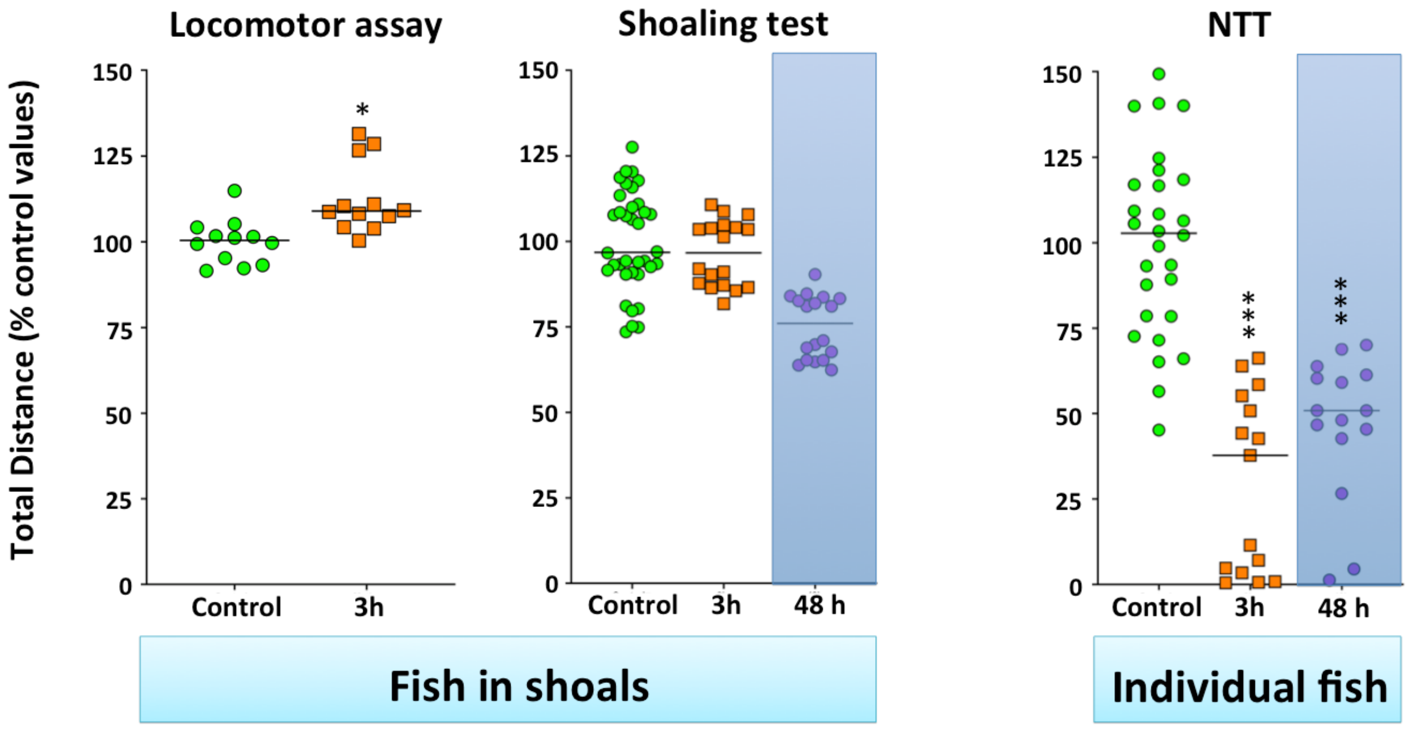

Supplementary Figure S6. Total distance moved during 6 min by the control and methamphetamine-exposed fish (40 mg/L) after 3 h of exposure. Moreover, for the shoaling test and novel tank test (NTT) the distance moved after 48 h of exposure is also presented. In the locomotor assay, the total distance was measured during the last 6 min of the 3 h exposure period of this assay. However, for the shoaling test and NTT, however, the locomotor activity was measured for 6 min immediately after the 3 h exposure period.

TH - AY644727

Carassius auratus tyrosine hydroxylase (TH) mRNA, partial cds

```
TH-F 5'-CAGCACACTGGTCAGCTCTC-3'
TH-R 5'-GCGATTTTTCTTGGAAACCA-3'

  1 agacgtttga agccaaaatc catcatcttg agaccagacc gagccgaaaa ccgaaggacg
 61 gtctggagga tctggagtat tacgttcagt gtgaggtgca cctttcagac gtcagcacac
121 tggtcagctc tctcaagaga agtgcagaga acgtcaaaac cactaaagag gtcaaatttc
181 actggtttcc aagaaaaatc gctgaactga ataaatgcca tcatctcatc actaaatttg
241 atccggatct ggatcaggat catccaggat tcactgaccc catttacaga aaacgccgga
301 agatgattgg agacattgcc ttcaaataca aacatggaga accgattccc agagtggaat
361 atacagaaga ggagatcgaa acatggcgtg aggtctactc caccctcagg gatttgtaca
421 ccacccatgc ttgcagtgaa catctagagg ctttctgctt actggagaaa cactgcggct
481 acagtcctga taacatccct cagctggagg aggtgtcccg ctt
```

```
cagcacactggtcagctctctcaagagaagtgcagagaacgtcaaaaccactaaagaggtcaaatttcactggtttc
caagaaaaatcgc
```

TH1 - NM_131149.1

Danio rerio tyrosine hydroxylase (th), mRNA – Length: 1470

```
AY644727      2  AGCACACTGGTCAGCTCTCTCAAGAGAAGTGCAGAGAACGTCAAAACCACTAAAGAGGTC  61
                 ||||| |||||||||||| | ||||||||||||||| | ||||||||||| || ||||||
NM_131149.1 400  AGCACGCTGGTCAGCTCTTTGAAGAGAAGTGCAGAGGATGTCAAAACCACAAAGGAGGTC  459

AY644727     62  AAATTTCACTGGTTTCCAAGAAAAATCGC  90
                 |||||||| |||||||| |||||||| ||
NM_131149.1 460  AAATTTCATTGGTTTCCCAGAAAAATAGC  488
```

Identities: 79/89 (89%)

Supplementary Figure S7. Homology between the amplified fragment of *Carassius auratus* tyrosine hydroxylase mRNA and *Danio rerio* tyrosine hydroxylase 1 (*th1*) mRNA

Supplementary Tables

Supplementary Table S1. List of primers used for the qPCR

Gene	ZFIN Acc number	GenBank Acc number		Sequence	Amplicon length
dbh	ZDB-GENE-990621-3	NM_001109694	FW	5'-TGCAACCAGTCCACAGCGCA	156 bp
			RV	5'-GCTGTCCGCTCGCACCTCTG	
gfap	ZDB-GENE-990914-3	NM_131373	FW	5'-GGATGCAGCCAATCGTAAT	97 bp
			RV	5'-TTCCAGGTCACAGGTCAG	
ppia2	ZDB-GENE-030131-8556	NM_212758.1	FW	5'-GGGTGGTAATGGAGCTGAGA	179 bp
			RV	5'-AATGGACTTGCCACCAGTTC	
slc6a3	ZDB-GENE-010316-1	NM_131755	FW	5'-AGACATCTGGGAAGGTGGTG	151 bp
			RV	5'-ACCTGAGCATCATACAGGCG	
slc18a2	ZDB-GENE-080514-1	NM_001256225.2	FW	5'-TGGAGCTCTGCAGCTTTTTGTGC	159 bp
			RV	5'-AACGCCGGCTCCAGCATAGC	
th1	ZDB-GENE-990621-5	NM_131149.1	FW	5'-GACGGAAGATGATCGGAGACA	95 bp
			RV	5'-CCGCCATGTTCCGATTTCT	
th2	ZDB-GENE-050201-1	NM_001001829.1	FW	5'-CTCCAGAAGAGAATGCCACATG	110 bp
			RV	5'-ACGTTCACTCTCCAGCTGAGTG	

Supplementary Table S2. Stability of methamphetamine in fish water under the experimental conditions of photoperiod and temperature

Nominal concentration (mg/L)	Time (h)	Measured concentration (mg/L)	Measured concentration (mean ± SE)
20	0	20.63<br>20.64<br>20.70	20.66±0.02
	24	20.35<br>20.82<br>20.72	20.63±0.14
	48	20.72<br>20.56<br>20.73	20.67±0.06
40	0	40.16<br>39.83<br>40.17	40.05±0.11
	24	41.93<br>39.90<br>39.23	40.35±0.81
	48	38.87<br>39.37<br>38.39	38.88±0.28

References

Green, J., Collins, C., Kyzar, E. J., Pham, M., Roth, A., Gaikwad, S., et al. (2012). Automated high-throughput neurophenotyping of zebrafish social behavior. *J. Neurosci. Methods* 210, 266–271. doi:10.1016/j.jneumeth.2012.07.017.

Kysil, E. V., Meshalkina, D. A., Frick, E. E., Echevarria, D. J., Rosemberg, D. B., Maximino, C., et al. (2017). Comparative Analyses of Zebrafish Anxiety-Like Behavior Using Conflict-Based Novelty Tests. *Zebrafish* 14, 197–208. doi:10.1089/zeb.2016.1415.
